# Identification of novel T cell proliferation patterns, potential biomarkers and therapeutic drugs in colorectal cancer

**DOI:** 10.7150/jca.91835

**Published:** 2024-01-01

**Authors:** Xu Wang, Shixin Chan, Longfei Dai, Yuanmin Xu, Qi Yang, Ming Wang, Qijun Han, Jiajie Chen, Xiaomin Zuo, Zhenglin Wang, Yang Yang, Hu Zhao, Guihong Zhang, Huabing Zhang, Wei Chen

**Affiliations:** 1 Department of General Surgery, The First Affiliated Hospital of Anhui Medical University, Hefei 230032, Anhui, China.; 2 Department of Gastroenterology, The First Affiliated Hospital of Wannan Medical College, Wuhu, 241000, China.; 3 Department of Dermatology, The First Affiliated Hospital of Anhui Medical University, Hefei 230032, Anhui, China.; 4 The Pathology Department of Anhui Medical University, Hefei 230032, Anhui, China.; 5 Department of Biochemistry and Molecular Biology, Metabolic Disease Research Center, School of Basic Medicine, Anhui Medical University, Hefei 230032, Anhui, China.; 6 The First Affiliated Chuzhou Hospital of Anhui Medical University, Chuzhou 239000, Anhui, China.

**Keywords:** T cell proliferation, colorectal cancer, prognosis, tumor microenvironment, chemotherapy, immunotherapy

## Abstract

**Background:** T cells are crucial components of antitumor immunity. A list of genes associated with T cell proliferation was recently identified; however, the impact of T cell proliferation-related genes (TRGs) on the prognosis and therapeutic responses of patients with colorectal cancer (CRC) remains unclear.

**Methods:** 33 TRG expression information and clinical information of patients with CRC gathered from multiple datasets were subjected to bioinformatic analysis. Consensus clustering was used to determine the molecular subtypes associated with T cell proliferation. Utilizing the Lasso-Cox regression, a predictive signature was created and verified in external cohorts. A tumor immune environment analysis was conducted, and potential biomarkers and therapeutic drugs were identified and confirmed via *in vitro* and *in vivo* studies.

**Results:** CRC patients were separated into two TRG clusters, and differentially expressed genes (DEGs) were identified. Patient information was divided into three different gene clusters, and the determined molecular subtypes were linked to patient survival, immune cells, and immune functions. Prognosis-associated DEGs in the three gene clusters were used to evaluate the risk score, and a predictive signature was developed. The ability of the risk score to predict patient survival and treatment response has been successfully validated using multiple datasets. To discover more possible biomarkers for CRC, the weighted gene co-expression network analysis algorithm was utilized to screen key TRG variations between groups with high- and low-risk. *CDK1*, *BATF*, *IL1RN*, and *ITM2A* were screened out as key TRGs, and the expression of key TRGs was confirmed using real-time reverse transcription polymerase chain reaction. According to the key TRGs, 7,8-benzoflavone was identified as the most significant drug molecule, and MTT, colony formation, wound healing, transwell assays, and *in vivo* experiments indicated that 7,8-benzoflavone significantly suppressed the proliferation and migration of CRC cells.

**Conclusion:** T cell proliferation-based molecular subtypes and predictive signatures can be utilized to anticipate patient results, immunological landscape, and treatment response in CRC. Novel biomarker candidates and potential therapeutic drugs for CRC were identified and verified using *in vitro* and *in vivo* tests.

## Introduction

Cancer has emerged as a prominent contributor to human mortality, as evidenced by the recorded incidence of more than 19 million recently diagnosed cancer cases and almost 10 million cancer-associated fatalities in 2020. Colorectal cancer (CRC) is the third most frequently occurring cancer and exhibits the second highest fatality rate, comprising approximately one-tenth of all cases of cancer and cancer-associated fatalities 1. Early-stage CRC can be cured by surgery; however, the majority of patients have advanced CRC, and recurrence and metastasis are often noticed, leading to poor clinical outcomes in patients with CRC 2-4. Overall, survival rates for patients with CRC have significantly improved owing to developments in traditional surgical techniques, chemotherapy, and treatment strategies. Programmed cell death protein 1 (*PDCD1*/*PD-1*) is an immunological checkpoint protein comprising 288 amino acids. *PD-1* is expressed on the surface of T cells and functions in apoptosis 5. Since the approval of the first type of *PD-1* inhibitor by the US Food and Drug Ministration (FDA) in 2014 and 2015 for the treatment of advanced lung cancer and melanoma, an increasing number of immune checkpoint inhibitors (ICIs) have been utilized to treat numerous types of malignant tumors. *PD-1* inhibitors have been shown to improve prognosis in metastatic CRC patients with deficient mismatch repair (dMMR) or high microsatellite instability (MSI-H) 6. However, only a subset of patients with CRC has benefited considerably from ICI therapy. To address this issue, more biomarker candidates have been recognized, including tumor burden mutations (TMB) and neoantigen load (NAL), but the prognostic capacity of these approaches is restricted due to their small percentage populations or moderate efficiency 7-9. Thus, the development of new and promising biomarker candidates and medicinal drugs for CRC patients is imperative if existing CRC treatment strategies are to be improved.

Changes in the tumor microenvironment (TME) are typically associated with the incidence and progression of malignant tumors 10. The TME contains numerous cellular types, such as tumor, stromal, and immune cells 11. Among these cell types in the TME, natural killer (NK) cells, in addition to natural killer T (NKT) cells, are increasingly utilized in treating cancer. In addition, the use of T lymphocytes has been studied. T cells have unique anticancer localization characteristics, as they show direct effector activity and auxiliary functions by recruiting other components of the immune response. Furthermore, T lymphocytes can expand *in vitro* and establish memory compartments, which are major features of antitumor monitoring 12. Previous studies have suggested that *CD4*+ and *CD8*+ T cell infiltration into malignant tumors not only represents the ongoing anti-tumor response by the host but also correlates with the prognosis of patients with cancer 13,14. Recent research 15 discovered 33 synthetic drivers of T cell proliferation via genome-wide large-scale screening; however, the effects of these T cell proliferation-related genes (TRGs) on CRC remain largely unknown. Understanding the effects of TRGs on cancer is vital for the development of new treatment strategies. Therefore, the relationship between these T-cell proliferation-related genes and patient prognosis and response to immunotherapy should be further investigated.

Advances in high-throughput sequencing technology have allowed investigators to utilize multiple categories of sequencing data acquired from free access databases such as The Cancer Genome Atlas (TCGA) and the Gene Expression Omnibus (GEO). Recently, numerous researchers have begun to use sequencing data from publicly available databases to identify new biomarkers for diagnosis and treatment, and to construct prognostic models or molecular classifications to predict clinical outcomes and improve the effects of immunotherapy and chemotherapy against various cancer types. Chen et al. established a predictive score related to the immune system for head and neck squamous cell carcinoma (HNSCC) 16. It can accurately predict the clinical outcomes, immune escape, and ICI benefits in patients diagnosed with HNSCC. Zhang et al. 17 used RNA N6-methyladenosine-related genes to categorize patients with gastric cancer into different molecular subtypes and calculated a predictive index to predict survival and immunotherapy response in gastric cancer. Six immune classifications among various cancer types were determined in a previous investigation using TCGA data 18: wound healing, interferon (IFN)-γ dominance, inflammation, lymphocyte depletion, immunologically quiet, and transforming growth factor-β dominance. The six immune classifications have been found to be related to the outcomes and immunological features of patients with cancer.

This study aimed to evaluate the genetic alterations, predictive significance, and expression of TRGs. Taken together, based on the TRGs, we identified molecular subtypes and predictive signatures that possess the ability to accurately anticipate patient outcome, immune landscape, and treatment sensitivity, and screened potential therapeutic biomarker candidates and drug molecules for clinical application in the treatment of CRC.

## Materials and Methods

### Collection and Analysis of Transcriptional and Clinical Data

The Genomic Data Commons Data Portal (https://portal.gdc.cancer.gov, TCGA-COAD, and TCGA-READ projects) and GEO database (https://www.ncbi.nlm.nih.gov/geo/, ID: GSE39582, GSE17536, GSE17537, GSE29621, GSE38832, GSE45404, and GSE62080) were used to obtain transcriptional and clinical data for CRC patients from various publicly available datasets. iMvigor210 (http://research-pub.gene.com/IMvigor210CoreBiologies) is a group of urothelial carcinoma patients that contain complete follow-up information and immunotherapy effects after treatment with Programmed cell death 1 ligand 1 (*PD-L1*) blockade medication. TCGA-CRC, GSE39582, GSE17536, GSE17537, GSE29621, GSE38832, and iMvigor210 contain full data on overall survival (OS), which were used to construct and verify our molecular classifications and prognostic signature. Two datasets, GSE45404 and GSE62080, which contain patients who underwent fluorouracil-based adjuvant chemotherapy (ACT) and the iMvigor210 cohort, were used to assess the efficacy of the constructed signature in anticipating the response to ACT and immune checkpoint inhibitor therapy in CRC. Pan-cancer information from 32 tumor types was obtained from the USCS Xena website (http://xenabroswer.net/hub).

Fragments per kilobase million (FPKM) information from TCGA-CRC was converted into transcription per million (TPM) using The R studio program (v1.4.1106; R tools for Statistical Computing, Vienna, Austria). Information from the GEO datasets was retrieved from the GPL570 platform (Affymetrix Human Genome U133 Plus 2.0 Array). RNA-seq information was further log-2 transformed, and batch effects of the combined datasets (TCGA-CRC and GSE39582) were removed using the *sva* R package in the *ComBat* algorithm. Patients diagnosed with CRC who had incomplete clinical or follow-up information were excluded. A flowchart of this investigation was drawn using Figdraw (www.figdraw.com).

### Genetic and Transcriptional Alterations to TRGs in CRC

Thirty-three T-cell proliferation-related genes were identified in a recent study 15. TCGA database was utilized to retrieve transcriptional mutation information, which was subsequently analyzed to determine the frequency of changes in copy number and the corresponding location data for the 33 TRGs. Gene Ontology (GO) and Kyoto Encyclopedia of Genes and Genomes (KEGG) analyses were done to in-depth study TRG-related biological functions and pathways utilizing R tools: “*ggplot2”*, “*Bioconductor”*, as well as “*org.Hs.eg.db”*. The Wilcoxon signed-rank test in the *limma* tool was used to compare TRGs expression between normal and tumor tissues. Moreover, the prognostic value and interactions among TRGs were assessed using KM and univariate Cox regression analyses.

### Consensus Clustering to Detect TRG Clusters

Consensus clustering was performed to determine molecular classifications based on the expression values of the TRGs. By increasing the clustering variable k, the categorization exhibiting the most intragroup connections and the fewest intergroup connections was determined. Then, principal component analysis (PCA) was utilized to differentiate the two identified molecular classifications using the *stats* R tool. The KM method was used to examine the variations in survival time among TRG clusters and was compared using the log-rank test and the *survival* and *survminer* R tools. A comparison was made between the clinical variables of the TRG clusters, and differentially expressed genes (DEGs) between the two clusters were found using the criteria |log fold-change| > 1 and *p* < 0.05. Immunological cell infiltration and immunological-associated pathways in the TRG clusters were identified via gene set variation analysis (GSVA) and single-sample gene set enrichment analysis (ssGSEA) using the *gsva* R package. The expression of three well-known immune checkpoint genes, including* PD-1*, programmed cell death 1 ligand 1 (*PD-L1*), and cytotoxic T-lymphocyte-associated protein 4 (*CTLA-4*) in the TRG clusters was examined using the Wilcoxon signed-rank test and represented using violin plots. Uni-variate Cox regression analysis was used to identify the prognosis-related DEGs (PRDEGs).

### Classifying Patients into Gene Clusters Based on DEGs between TRG Clusters

PRDEGs between TRG clusters were used to perform consensus clustering to classify patients with CRC into three distinct groups. Clinical features and TRG expression in the three gene clusters were analyzed using heatmaps, boxplots, and Wilcoxon signed-rank test. Survival times for gene clusters were analyzed utilizing KM approach, and the log-rank test was performed to examine them. *PD-1*, *PD-L1*, and *CTLA-4* expression in the three gene clusters was also analyzed.

### Creation and Validation of the T Cell Proliferation-related Prognostic Signature

DEGs were identified among three gene clusters. Based on these DEGs, least absolute shrinkage and selection operator (LASSO) regression and multi-variate cox regression analyses were performed to identify genes that could be used to construct a predictive signature utilizing the *survival*, *survminer*, and *glmnet* R packages. The formula used to calculate the risk score was as follows:

Risk score = 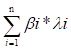


where n is the number of genes used in the signature construction and and express the regression coefficient and gene expression, respectively. Based on the risk score, individuals diagnosed with CRC were stratified into high- and low-risk groups. The study assessed the relationships between risk score, survival time, and status. Additionally, both univariate and multivariate Cox regression analyses were performed to identify the unique predictive variables in CRC patients using the risk score along with additional relevant clinical features. The efficiency of the T cell proliferation-related prognostic signature for predicting CRC patient survival was further verified in the training cohort and five independent cohorts (GSE17536, GSE17537, GSE29621, GSE38832, and iMvigor210) using the KM and receiver operating characteristic (ROC) methods. The C-index of the signature was calculated and compared to ten other published signatures for CRC 19-28. Gene sets related to angiogenesis, epithelial to mesenchymal transition (EMT), and the cell cycle were obtained from a previous study 29, and these gene sets were applied to the z-score technique using the *gsva* R program. Moreover, the relationship between the risk score and z-score associated with malignant biological processes in pan-cancer was evaluated using Pearson's correlation analysis.

### TME, MSI, Tumor Mutation Burden (TMB), and Cancer Stem Cell (CSC) Index Differences of the High- with Low-risk Groups

The CIBERSORT algorithm was used to quantify infiltrating immunological cells in the CRC samples, and the Spearman technique was used to assess the association between the risk score and immunological cell abundance. The relationship between immunological cells and the 10 signature genes was also analyzed. The Wilcoxon signed-rank test was used to compare the variations in TME scores, such as stromal, immune, and ESTIMATE scores, between the high- and low-risk groups, and violin plots were used to visualize them. The TMB score, MSI status, and CSC index in both risk groups were examined using the Wilcoxon signed-rank test and the Spearman technique.

### Immune Checkpoints Expression, Tumor Immune Dysfunction and Exclusion (TIDE) Score, and Immune Cell proportion Score (IPS) in both High- and Low-risk Groups

To assess the efficacy of the risk score in anticipating patient responses to ICI therapy, the expression of immunological checkpoint genes in the low- and high-risk groups was compared. The TIDE scores for patients were obtained from the TIDE website (https://tide.dfci.harvard.edu/), and a comparison was performed on the scores for the two risk groups to identify the likelihood of tumor immune escape. The IPSs for the CRC samples were retrieved from The Cancer Immunome Atlas (TCIA, https://tcia.at/) and were utilized to anticipate patient response to different ICI therapies, such as *PD-1*/*PD-L1*/*PD-L2, CTLA-4, CTLA-4, and PD-1*/*PD-L1*/*PD-L2* blockers. In addition, a comparison was made between the IPSs of CRC samples in the high- and low-risk groups. Moreover, the clinical application of risk scores to predict ICI responses was explored using the iMvigor210 cohort to calculate complete response (CR), partial response (PR), stable disease (SD), and progressive disease (PD) values.

### Association of Risk Score with IC_50_ of Therapeutic Medicines

IC_50_ is defined as the half-maximal inhibitory concentration of a therapeutic drug necessary to achieve 50% suppression of cancer cells. The IC_50_ values of various therapeutic drugs, including 5-fluorouracil, were compared between the high- and low-risk groups using the pRRophetic R tool. In the GSE45404 and GSE62080 datasets, patients diagnosed with CRC were subjected to fluorouracil-based ACT, and the risk scores between the non-response (NR) and response (R) groups in these two datasets were compared to measure the effectiveness of the risk score in anticipating patient responses to ACT.

### WGCNA Algorithm to Identify Key TRGs and for *in vitro* Validation via PCR

WGCNA was performed to identify key TRGs according to the risk groups and the intersections among the identified 33 TRGs. A suitable power exponent was utilized to transform the adjacency matrix (AM) into a topological overlap matrix. Correlation analyses were performed to screen for the key modules that were most relevant to the risk groups. *P*-values were used to identify the most significant modules, and the intersection genes between these modules and 33 TRGs were defined as key TRGs. Expression levels of four key TRGs in different single cell types were evaluated using GSE108989 and GSE146771 datasets via TISCH database (http://tisch.comp-genomics.org/). qRT-PCR was performed on a normal colon cell line (NCM-460) and four CRC cell lines (HT-29, HCT-116, SW-480, and RKO) to validate the expression of key TRGs in CRC. Total RNA was extracted using TRIzol reagent (Life Technologies, Carlsbad, CA, USA), and complementary DNA (cDNA) was synthesized using a PrimeScript RT kit (Vazyme, Nanjing, China). The amount of cDNA was evaluated using TB Green Premix Ex Taq II (GenStar, Guangdong, China) and a LightCycler480 System (Applied Biosystems, Waltham, MA, United States). The relative expression levels of the four key TRGs were measured using the 2^-ΔΔCt^ approach and normalized to that of GAPDH. The expression levels in different cell lines were compared using t-tests. The following primer sequences were used for the key TRGs: *CDK1*, forward: 5'-AAACTACAGGTCAAGTGGTAGCC-3', reverse: 5'-TCCTGCATAAGCACATCCTGA-3'; *BATF*, forward: 5'-TATTGCCGCCCAGAAGAGC-3', reverse: 5'-GCTTGATCTCCTTGCGTAGAG-3'; *IL1RN*, forward: 5'-CATTGAGCCTCATGCTCTGTT-3', reverse: 5'-CGCTGTCTGAGCGGATGAA-3'; *ITM2A*, forward: 5'-ATCCTGCAAATTCCCTTCGTG-3', reverse: 5'-CAGGTAAGCAGTCATTCCCTTT-3'; and *GAPDH*, forward: 5'-GGGAAGGTGAAGGTCGGAGT-3', reverse: 5'-GGGGTCATTGATGGCAACA-3'.

### Screening Possible Therapeutic Drugs Based on Key TRGs

To screen for possible medicinal drugs according to the four key TRGs, a list of drug molecules was determined using the Drug Signatures Database (DSigDB) via the Enrichr online website (https://maayanlab.cloud/Enrichr/). According to the adjusted *p*-value, the eight most significant drug molecules were identified as potential therapeutic drugs. Three-dimensional (3D) structures of the eight drug molecules were obtained from the PubChem website (https://pubchem.ncbi.nlm.nih.gov/).

### Cell Culture

Normal human intestinal epithelial and CRC cell lines (NCM-460, HT-29, and HCT-116) were acquired from the American Typical Culture Center. Cells were incubated in Dulbecco's modified Eagle's medium (DMEM) supplemented with 10% fetal bovine serum (FBS; Lonsera, Austria) and 1% double antibody (streptomycin and penicillin) at 37 °C with 5% CO_2_.

### Western Blotting

After treating with 50μM 7,8-benzoflavone for 48 hours, protein extraction was conducted utilizing RIPA buffer (Beyotime, China) supplemented with protease and phosphatase inhibitors in HT-29 and HCT-116 cell lines. The Western blotting procedures adhered to established protocols as delineated in prior publications 30. The primary antibodies, anti-*CDK1*, anti-*IL1RN* and anti-*GAPDH*, were procured from Zenbio, China.

### Cell Viability Assay

In 96-well plates, HT-29 and HCT-116 cells were cultivated at a density of 3 × 10^3^ cells/well and each treatment consisted of six replicates. Following cell adhesion to the plate wall, the experimental groups were subjected to a predesigned 7,8-benzoflavone concentration gradient (10, 25, 50, 75, 100, 125, 150, 175, and 200 μM), whereas the control group was treated with dimethyl sulfoxide (DMSO) at the same concentrations. Following a 48h incubation at 37 °C, 25 μL MTT solution was applied to all wells, and the samples were incubated for 1 h. Then, 100 μL DMSO was added to all wells. Five minutes later, a microplate analyzer was used to evaluate absorbance at 490 nm in each well. Differences in OD values at all concentrations were compared, and IC_50_ values were calculated using the GraphPad Prism software (version 9.4).

### Colony Formation Assay

HT-29 and HCT-116 cells were cultured in 6-well plates at a density of 5 × 10^3^ per well. The experimental and control groups were treated with 7,8-benzoflavone at a concentration of 50 μM or an equal amount of DMSO. All samples were incubated in DMEM supplemented with 10% FBS and 1% double antibody. Each three days, the DMEM was refreshed, and 7,8-benzoflavone and DMSO were added to each well to maintain the drug concentration. After incubation for 10 days, adherent cells were preserved using methanol for 10 min and stained with 0.1% crystal violet for 15 min. Finally, each well was washed twice with peripheral blood smear (PBS). The number of clones was counted using ImageJ software and compared using t-tests and GraphPad Prism software (n = 3).

### Wound Healing Assay

In 6-well plates, HT-29 and HCT-116 cells were seeded at a 1.5 × 10^6^ cells/well. Following cellular adherence to the surface of 6-well plates, a scratch was made using the tip of a 200 μL pipette and the experimental and control groups were treated with 7,8-benzoflavone at a concentration of 50 μM or an equal amount of DMSO, respectively. The cells were incubated in DMEM containing 2% FBS. Scratches were observed and photographed using a microscope at 0, 24, and 48 h after washing with PBS. Cell migration distances after 48 h were calculated using ImageJ software and compared using t-tests and GraphPad Prism (n = 3).

### Transwell Assay

A suspension of 5 × 10^4^ CRC cells, treated with or without 7,8-benzoflavone, was prepared in 200 μL of serum-free DMEM and subsequently introduced into the top chamber of the Transwell. DMEM (600 μL DMEM with 10% FBS) was then applied to the lower compartments. Following 48h incubation, the cells situated in the lowermost part of the membrane were treated with methanol for fixation, followed by staining with 0.1% crystal violet. Five randomized versions of each well were imaged under a microscope. Relative cell counts were calculated using ImageJ and compared using t-tests and GraphPad Prism software (n = 5).

### Nude mouse tumor formation assay

Five-week-old nude male BALB/c mice were obtained from the Model Animal Research Center of Nanjing University and housed in a specific pathogen-free (SPF) environment. The experimental protocol involving animal subjects was approved by the Animal Ethical Committee of Anhui Medical University, and all animal testing procedures were strictly conducted in compliance with the instructions prescribed by the Animal Center of Anhui Medical University. All experiments involving animals were conducted under the instructions of the ARRIVE Guidelines 2.0, the U.K. Animals (Scientific Procedures) Act, 1986 and associated guidelines. To induce tumorigenesis, 2 × 10^6^ HCT116 cells were subcutaneously inoculated into the right flank of nude mice and monitored for tumor development. Tumor volume was evaluated as 1/2 × length × width × height. Once the tumor volume reached 100 mm^3^, experimental mice received 50 mg/kg 7,8-Benzovlavone (solubilized in a solution comprising 10% DMSO, 10% Tween80, in addition to 80% NaCl) via injection into the tumor each two days, while control mice received an equivalent volume of vehicle. After 4 weeks, the mice were euthanized and the tumors were harvested for subsequent examination.

### Immunofluorescence staining

To prepare the colonic tumor tissues from nude mice for histological analysis, The specimens were initially immersed in a solution of 10% formalin and subsequently immersed in paraffin. To prepare colonic tumor tissues from nude mice for histological analysis, the specimens were initially immersed in a solution of 10% formalin and subsequently immersed in paraffin. Subsequently, the tissue blocks were sliced into 5 μm slices and affixed onto adhesive slides. Subsequently, the slides were deparaffinized using xylene and ethanol and treated with distilled water. To retrieve antigens, sections were subjected to EDTA antigen repair. Subsequently, the slides were incubated at 37 °C for 30 min with 5% normal goat serum to prevent non-specific binding sites from occupying. The slides were incubated overnight at 4 °C after addition of the primary antibody for *Ki67* (Abclonal, 1:50). After washing, the slides were incubated with secondary antibody in the dark at 37 °C for 1h. Slices were counterstained with DAPI at room temperature in the dark for 10 min, followed by microscopic examination.

## Results

### Genetic and Transcriptional Changes to TRGs in CRC

**Figure 1:**
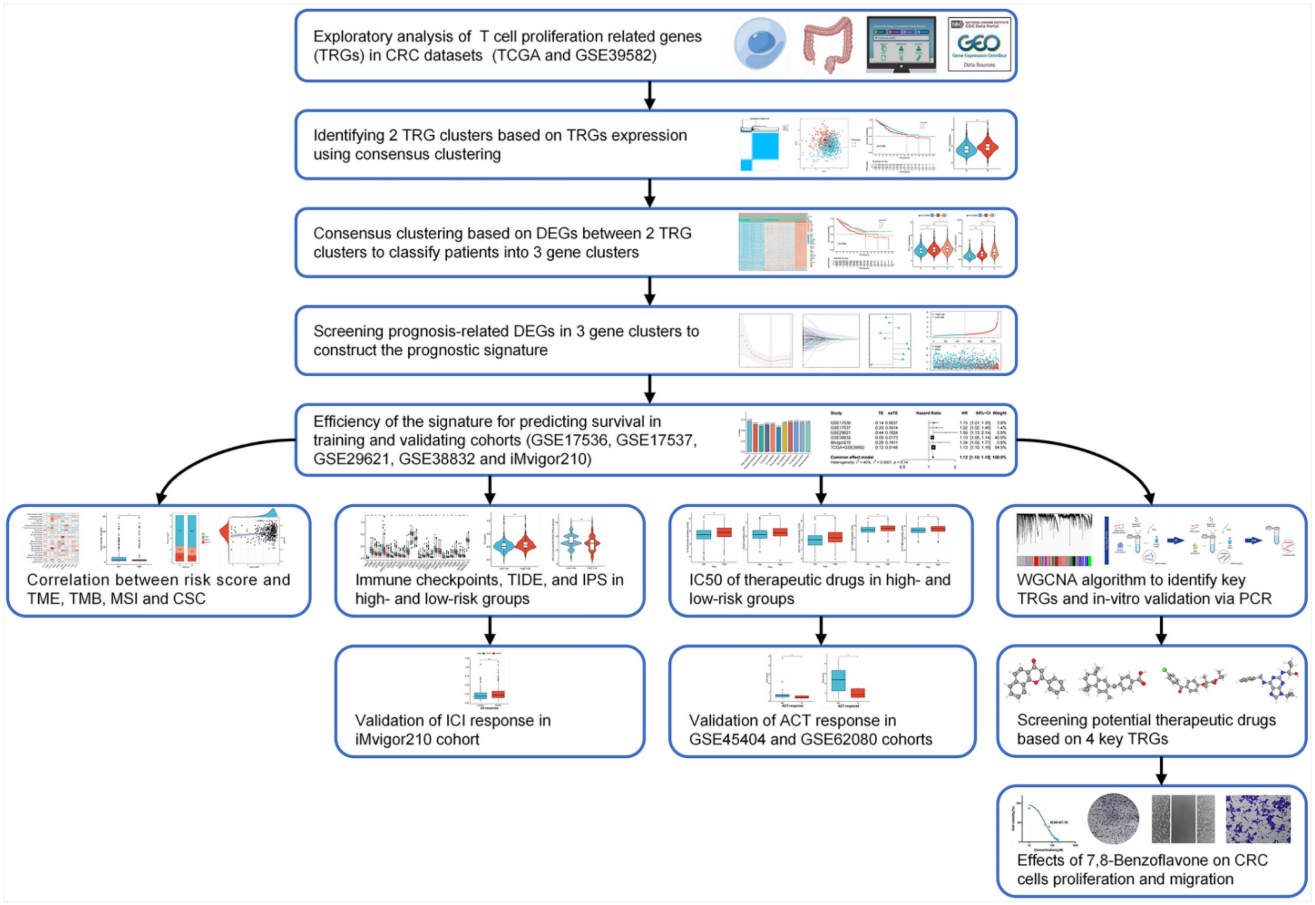
Workflow of the present study.

**Figure 2:**
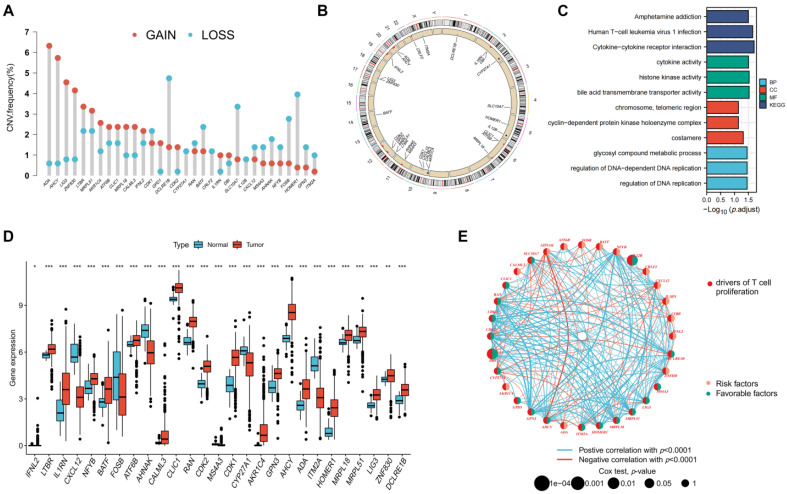
Genetic and transcriptional changes to TRGs in CRC. **(A)** CNV of TRGs in CRC. **(B)** The chromosomal positions of CNVs within TRGs in humans. **(C)** Functional analyses of TRGs using GO and KEGG. **(D)** Twenty-seven TRGs were differentially expressed (*p* < 0.05); *CXCL12*, *FOSB*, *AHNAK*, *MS4A3*, *CYP27A1*, and *ITM2A* were downregulated, whereas the others were upregulated. **(E)** The interactions between the TRGs and their predictive significance were revealed by constructing a network. *p < 0.05; **p < 0.01; ***p < 0.001.

A flowchart of the investigation is shown in **Figure 1**. **Table S1** shows the clinical data of the patients in all datasets. A recent study determined thirty-three TRGs (**Table S2**). The copy number variation (CNV) of TRGs in CRC was investigated (**Figure 2A**). *ADA*, *AHCY*, *LIG3*, *ZNF830*, *LTBR*, and *MRPL51* showed widespread CNV increases, whereas *DCLRE1B*, *BATF*, *SL10A7*, *FOSB*, and *HOMER1* showed CNV reductions. **Figure 2B** illustrates the chromosomal positions of CNVs within TRGs in humans, and the incidence of somatic mutations in TRGs in patients was also measured (**Figure S1**). Then, GO and KEGG analyses were performed to identify significant biological processes (BP), cellular components (CC), molecular functions (MF), and pathways (**Figure 2C**). The present TRGs were primarily associated with the BP of glycosyl compound metabolic processes, control of DNA-dependent DNA replication, and control of DNA replication. TRGs were correlated with the CC of chromosomes, telomeric regions, the cyclin-dependent protein kinase holoenzyme complex, and costameres, which are also implicated in the MF of cytokine activity, histone kinase activity, and bile acid transmembrane transporter activity. These TRGs participate in several pathways, including amphetamine addiction, human T-cell leukemia virus 1 infection, and the cytokine-cytokine receptor interactions. Among these, 27 TRGs were differentially expressed (*p* < 0.05); *CXCL12*, *FOSB*, *AHNAK*, *MS4A3*, *CYP27A1*, and *ITM2A* were downregulated, whereas the others were upregulated (**Figure 2D**). The interactions between the TRGs and their predictive significance were revealed by constructing a network (**Figure 2E**). The survival curves of prognosis-associated TRGs are shown (**Figure S2**).

### Identification of TRG Clusters Using Consensus Clustering

**Figure 3:**
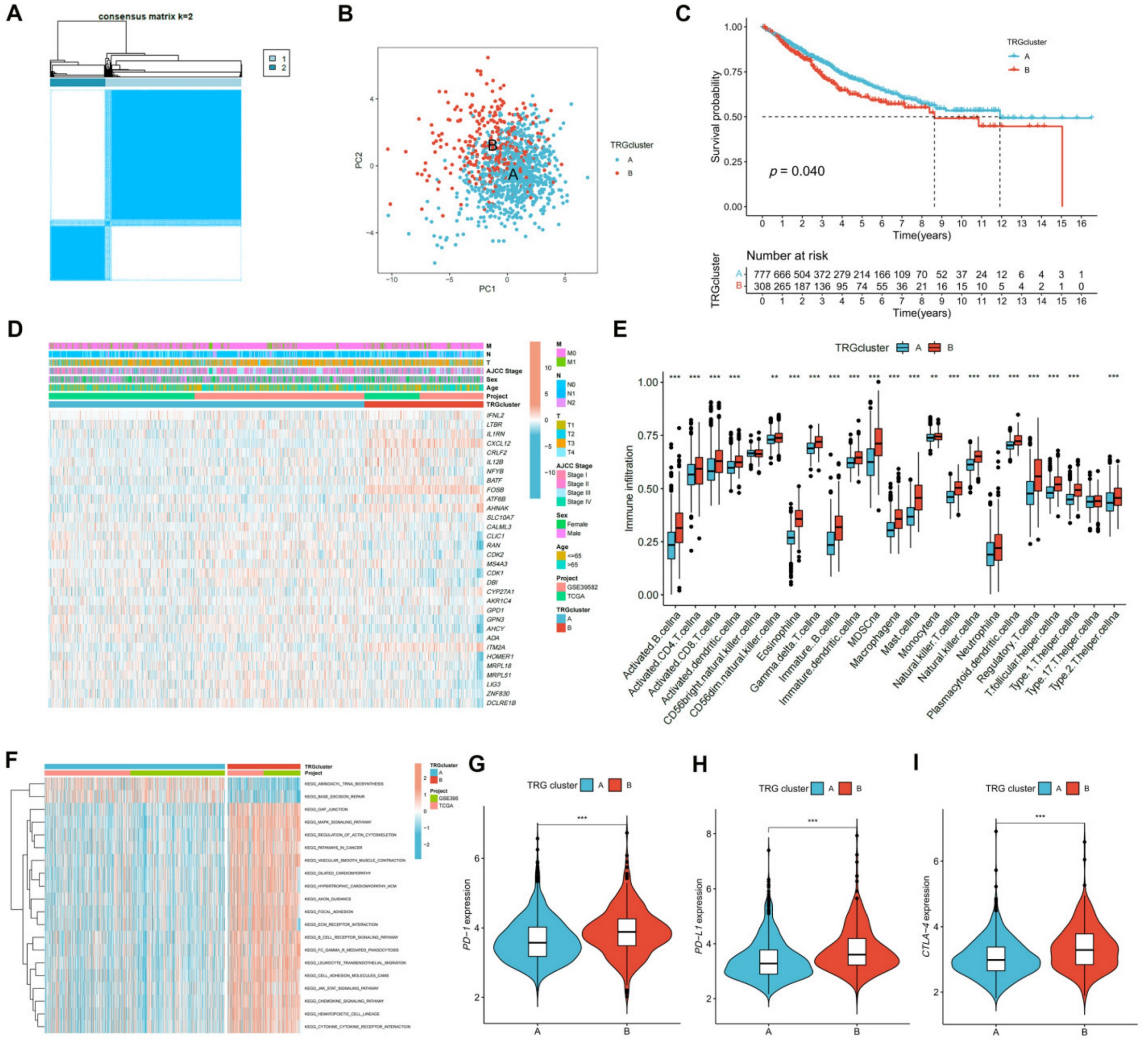
Identification of TRG clusters using consensus clustering. **(A)** The expression of the TRGs were used to perform a consensus clustering analysis and the patients with CRC were divided into two TRG clusters. **(B)** Satisfactory separation between the two TRG clusters was observed after the PCA. **(C)** The KM curve showed that patients with TRG cluster A had a more favorable prognosis than those with TRG cluster B (*p* = 0.040); **(D)** The relationship between the clinical characteristics, TRG cluster, and TRG expression is presented in a heatmap. **(E)** The ssGSEA results revealed that TRG cluster B had greater immune cell infiltration levels than TRG cluster A. **(F)** TRG cluster B was enriched in cancer-associated pathways pathways. **(G-H)** Expression levels of *PD-1*, *PD-L1*, and *CTLA-4* in two clusters. **p < 0.01; ***p < 0.001.

The expression of the TRGs were used to perform a consensus clustering analysis and the patients with CRC were divided into two TRG clusters (**Figure 3A; Figure S3**). Satisfactory separation between the two TRG clusters was observed after the PCA (**Figure 3B**). The KM curve showed that patients with TRG cluster A had a more favorable prognosis than those with TRG cluster B (**Figure 3C**). The relationship between the clinical characteristics, TRG cluster, and TRG expression is presented in a heatmap (**Figure 3D**). The ssGSEA results revealed that TRG cluster B had greater immune cell infiltration levels than TRG cluster A (**Figure 3E**). The GSEA demonstrated that the TRG cluster A exhibited enrichment in the pathways for aminoacyl tRNA biosynthesis and base excision repair. In addition, some cancer-associated pathways, such as the MAPK signaling pathway, were enriched in TRG cluster B (**Figure 3F**). Furthermore, TRG cluster B showed a greater expression of immune checkpoint genes, which includes *PD-1* (**Figure 3G, *p* < 0.001**), *PD-L1* (**Figure 3H, *p* < 0.001**), and* CTLA-4* (**Figure 3I, *p* < 0.001**).

### Classification of Patients into Gene Clusters on the basis of DEGs between TRG Clusters

**Figure 4:**
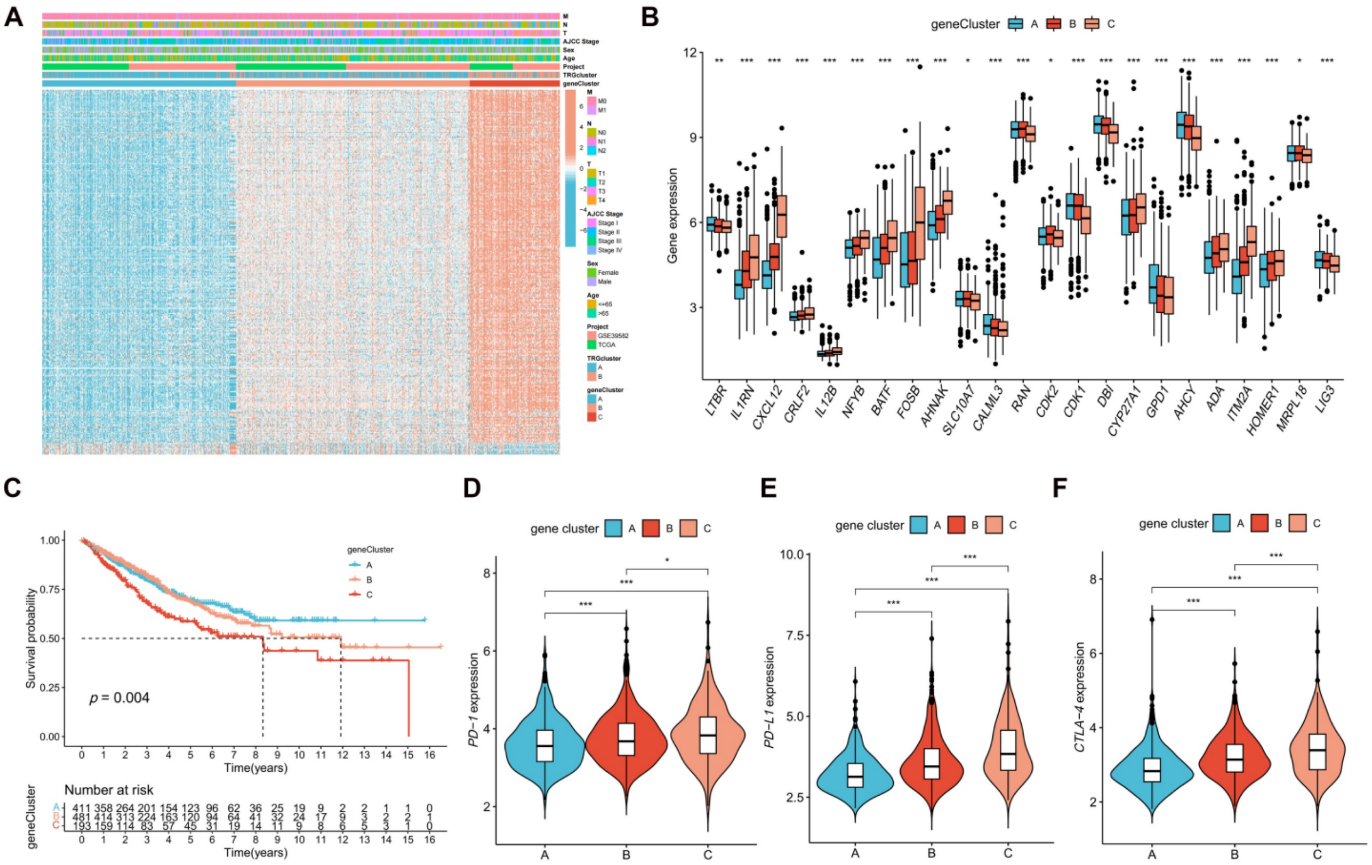
Identification of gene clusters based on DEGs. **(A)** Heatmap showed the association between three gene cluster and clinical features. **(B)** Expression levels of DETRGs in three gene clusters. **(C)** The KM curve shows that patients in cluster A had the longest survival time, whereas patients in cluster C had the worst prognosis (*p* = 0.004). **(D-F)** Expression levels of *PD-1*, *PD-L1*, and *CTLA-4* in three gene clusters. *p < 0.05; **p < 0.01; ***p < 0.001.

The DEGs between TRG clusters A and B were determined, and their expression profiles were used to divide the patients with CRC into three gene clusters** (Figure S4)**. The associations between clinical characteristics, TRG clusters, gene clusters, and DEGs are shown in Figure 4A. DEG expression in TRGs is shown in a boxplot **(Figure 4B)**. In addition, the overall survival time of patients with CRC within the three gene clusters was analyzed. The KM plot revealed that gene cluster B had the most favorable prognosis in the first four years, whereas gene cluster A exhibited the most favorable prognosis after five years. However, gene cluster C showed the shortest survival time at all time points **(Figure 4C, *p* = 0.004)**. Furthermore, gene cluster A had the lowest expression of *PD-1* **(Figure 4D, *p* < 0.001)**, *PD-L1* **(Figure 4E, *p* < 0.001)**, and *CTLA-4* **(Figure 4F, *p* < 0.001)**, whereas immune checkpoints showed the highest expression in cluster C.

### Creation and Validation of the T Cell Proliferation-related Predictive Signature

**Figure 5:**
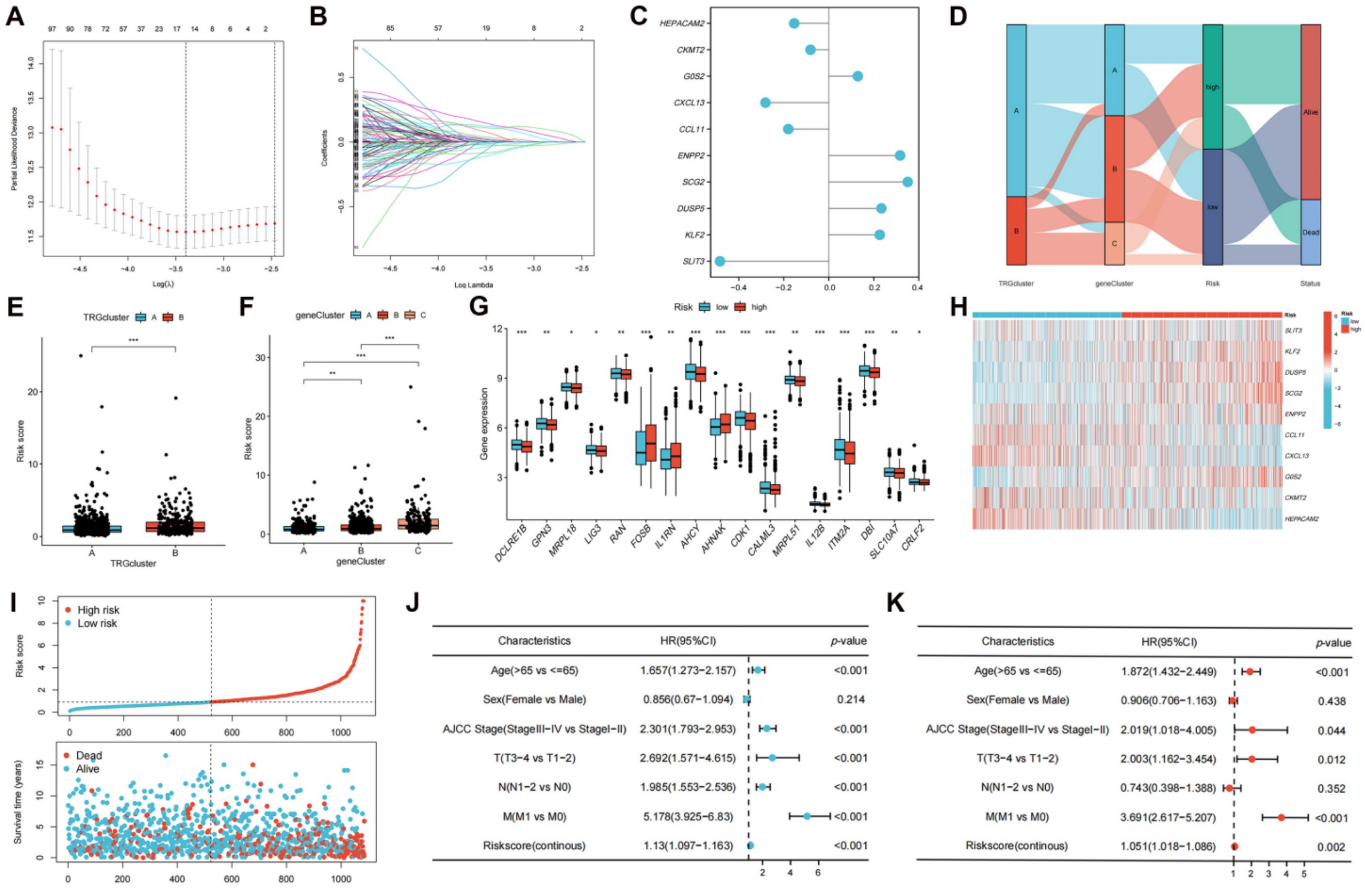
Development of the TRGs-related prognostic signature. **(A-B)** LASSO and stepwise Cox analyses were performed to monitor PRDEGs that may be utilized to construct the predictive signature. **(C)** 10 signature genes were screened, and the coefficient values of the multivariate Cox regression are shown. **(D)** The Sankey diagram depicts the correlation between the risk score, TRG cluster, gene cluster, and survival status of patients diagnosed with CRC. **(E-F)** The risk scores for the two TRG clusters and three gene clusters are shown in the boxplots. **(G)** Differentially expressed TRGs between high- and low-risk groups are shown. **(H)** The expression differences of the 10 signature genes. **(I)** Patients with high-risk CRC exhibited a higher risk of mortality. Univariate **(J)** and multivariate **(K)** analyses demonstrated that the risk score may be utilized as a distinct predictive variable for patients with CRC. *p < 0.05; **p < 0.01; ***p < 0.001.

**Figure 6:**
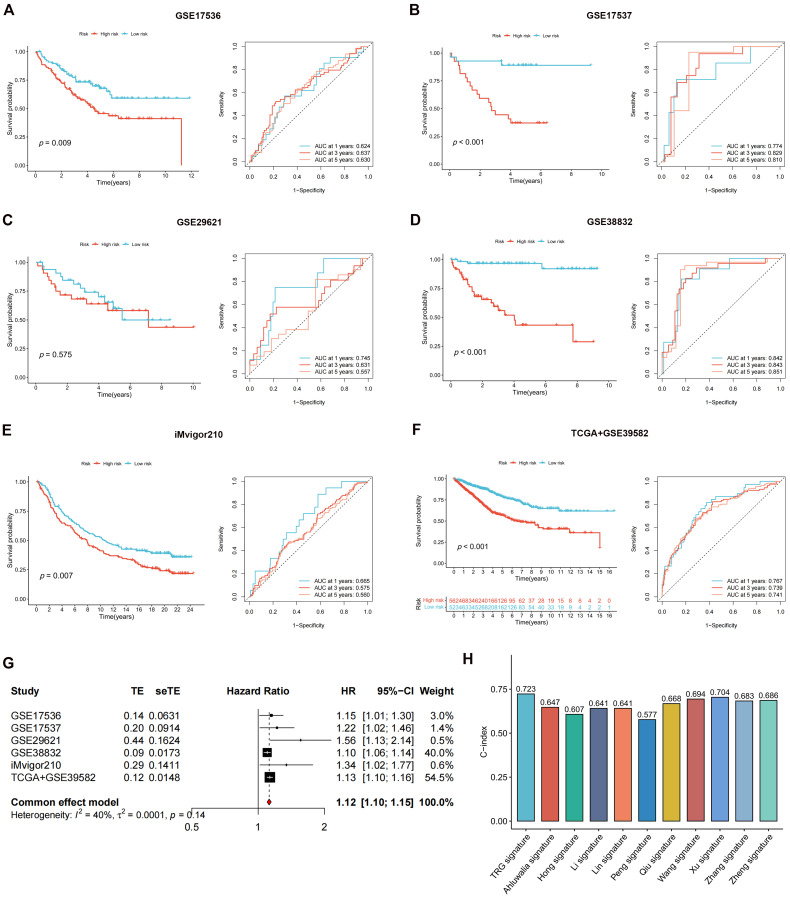
Validating the accuracy of the risk score in predicting patient outcome. **(A-F)** The results for five independent cohorts showed that patients with low-risk CRC exhibited a significantly longer survival duration than patients diagnosed with high-risk CRC and that the risk index had the ability to accurately anticipating patient survival. **(G)** The meta-analysis results did not show evidence of heterogeneity between the training and validation cohorts. **(H)** C-index of our signature showed a superior performance at predicting CRC prognosis compared with other 10 published signatures.

**Figure 7:**
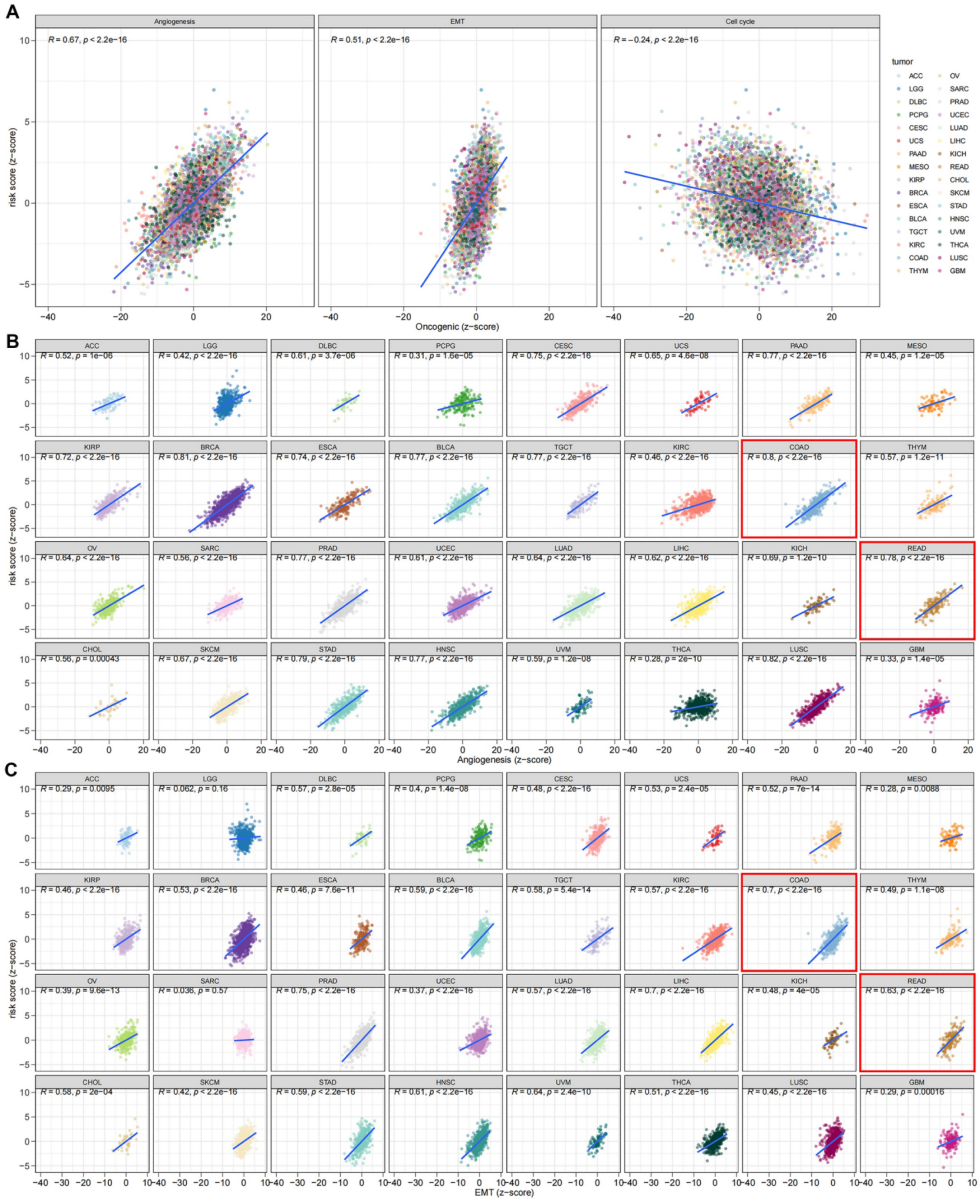
The risk score was correlated with malignant features in pan-cancer **(A)** and most of the tumor types **(B-C)**.

The DEGs among the gene clusters were further identified, LASSO and stepwise Cox analyses were performed to monitor PRDEGs that may be utilized to construct the predictive signature, and 10 signature genes were screened (*SLIT3*, *KLF2*, *DUSP5*, *SCG2*, *ENPP2*, *CCL11*, *CXCL13*, *G0S2*, *CKMT2*, and *HEPACAM2*). The processes for LASSO regression are shown in **Figure 5A-B** and the coefficient values of the multivariate Cox regression are shown** (Figure 5C; Table S3)**. The risk score was measured in accordance with the signature gene expression and coefficient values. Patients with CRC were divided into high- and low-risk groups based on their risk score values. The Sankey diagram depicts the correlation between the risk score, TRG cluster, gene cluster, and survival status of patients diagnosed with CRC** (Figure 5D)**. The risk scores for the two TRG clusters **(Figure 5E)** and three gene clusters** (Figure 5F)** are shown in the boxplots. Differentially expressed TRGs are shown in **Figure 5G** and the expression differences of the 10 signature genes are shown in the heatmap** (Figure 5H)**. Patients with high-risk CRC exhibited a higher risk of mortality **(Figure 5I)**. Univariate **(Figure 5J, *p* < 0.001)** and multivariate **(Figure 5K,* p* = 0.002)** analyses demonstrated that the risk score may be utilized as a distinct predictive variable for patients with CRC. KM and area under curve (AUC) values were utilized to assess the efficacy of the risk score in predicting patient survival. The results for five independent validation cohorts, GSE17536 **(Figure 6A, *p* = 0.009, 1-year AUC = 0.624, 3-year AUC = 0.637, 5-year AUC = 0.630)**, GSE17537 **(Figure 6B, *p* < 0.001, 1-year AUC = 0.774, 3-year AUC = 0.829, 5-year AUC = 0.810)**, GSE29621 **(Figure 6C, *p* = 0.575, 1-year AUC = 0.745, 3-year AUC = 0.631, 5-year AUC = 0.557)**, GSE38832 **(Figure 6D, *p* < 0.001, 1-year AUC = 0.842, 3-year AUC = 0.843, 5-year AUC = 0.851)**, and iMvigor210 **(Figure 6E, *p* = 0.007, 1-year AUC = 0.665, 3-year AUC = 0.575, 5-year AUC = 0.560)**, showed that patients with low-risk CRC exhibited a significantly longer survival duration than patients diagnosed with high-risk CRC and that the risk index had the ability to accurately anticipating patient survival. For the training cohort (TCGA + GSE39582), according to the KM curve, low-risk patients exhibited less favorable outcomes **(*p* < 0.001)**, and the 1-, 3-, and 5-year AUC measures were 0.767, 0.739, and 0.741, respectively **(Figure 6F)**. The meta-analysis results did not show any evidence of heterogeneity between the training and validation cohorts **(Figure 6G)**. The C-index for the prognostic signature was calculated and compared to 10 published CRC signatures. These findings demonstrated that our signature was superior in predicting CRC prognosis** (Figure 6H)**. Malignant features of angiogenesis, EMT, and cell cycle were quantified using gene sets via the z-score algorithm, and positive correlations were observed between risk score and angiogenesis **(R = 0.67, *p* < 0.0001)** and EMT **(R = 0.51, *p* < 0.0001)** z-score, while risk score had an adverse association with cell cycle z-score **(R = -0.24, *p* < 0.0001)** in the overall pan-cancer cohort **(Figure 7A)**, and positive correlations were also observed in most of the separate cancer types **(Figure 7B-C)**.

### TME, Tumor Mutation Burden (TMB), Microsatellite Instability (MSI), and Cancer Stem Cell (CSC) Index between the High- and Low-risk Groups

**Figure 8:**
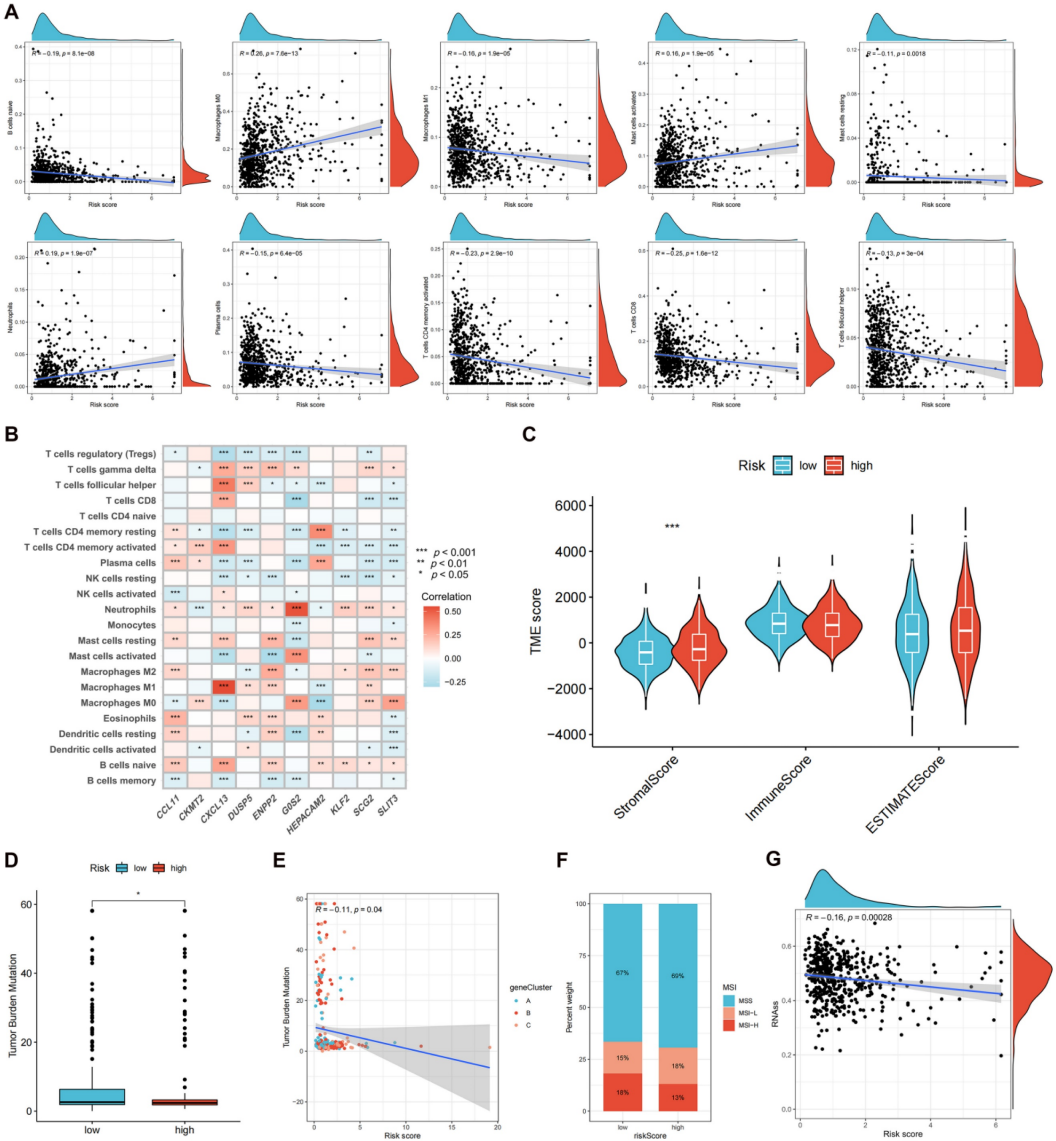
Evaluating the tumor microenvironment in two risk groups. **(A)** The correlation between the risk score and immune cell infiltration is shown. **(B)** The association of signature genes with immune cell abundance is shown. **(C)** Immune-related scores in two risk groups. TMB **(D-E)**, MSI **(F)**, and CSC **(G)** in high- and low-risk groups. *p < 0.05; **p < 0.01; and ***p < 0.001.

The correlation between the risk score and immune cell infiltration is shown in** Figure 8A**. The results revealed that 10 forms of immune cells are associated with the risk score: naive B cells, M0 and M1 macrophages, activated and resting mast cells, neutrophils, plasma cells, activated memory* CD4*+, *CD8*+, and follicular helper T cells. The association of signature genes with immune cell abundance is shown in** Figure 8B**. The high-risk group was associated with a greater stromal score** (Figure 8C)** and reduced TMB** (Figures 8D-E)**. Figure 8F shows the MSI status percentages in the two studied groups. The risk score was negatively associated with the CSC index **(Figure 8G)**.

### Immune Checkpoints Expression, TIDE Score, and IPS in the High- and Low-risk Groups

**Figure 9:**
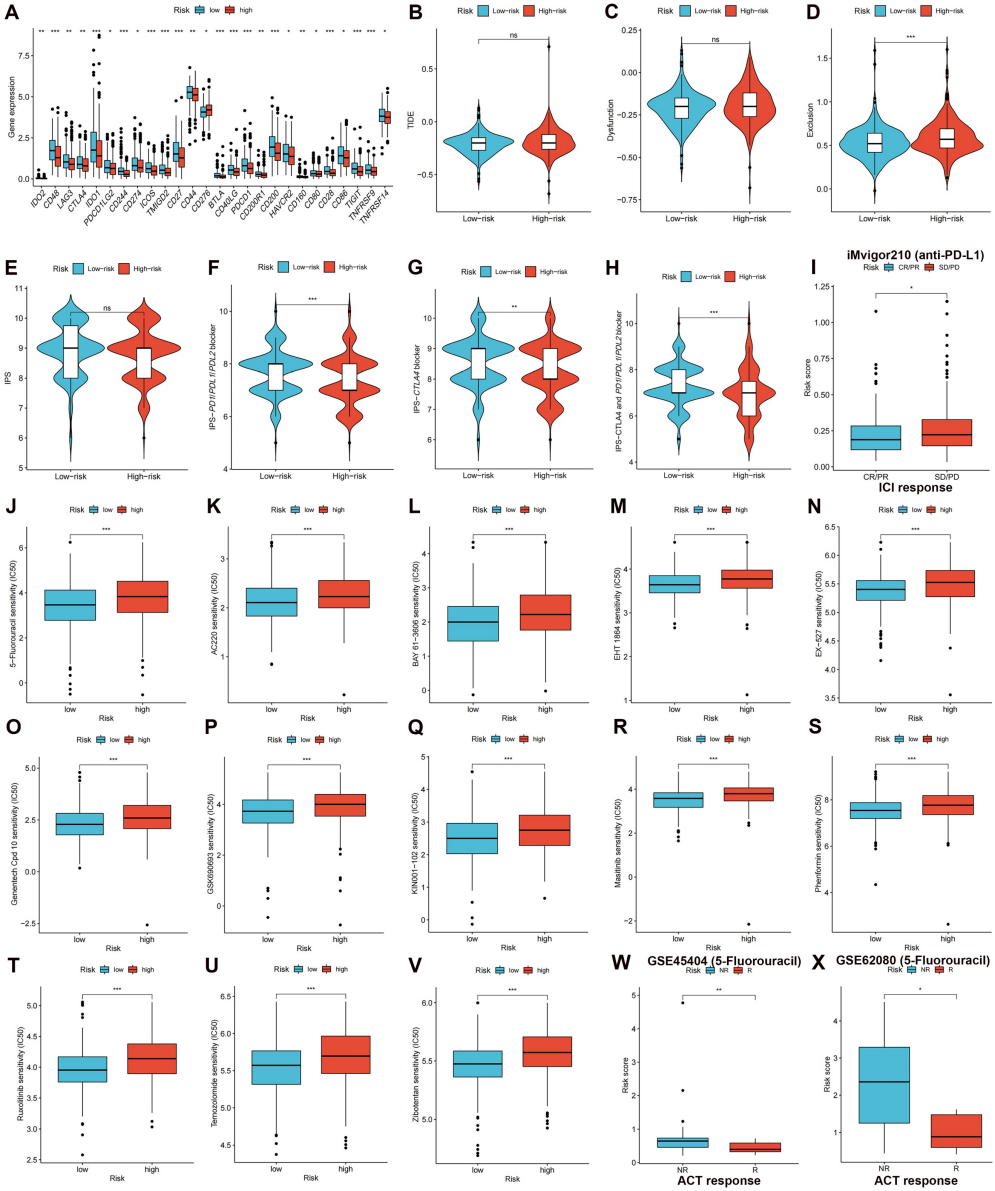
Validating therapeutic benefits in patients using the risk score. **(A)** The low-risk group displayed heightened expression patterns of immune checkpoint genes. **(B-D)** Predicted TIDE score in high- and low-risk groups. **(E-H)** IPSs in high- and low-risk groups are shown in violin plots. **(I)** Response to *PD-L1* blockade therapy between high- and low-risk groups. **(J-V)** Low-risk group had higher sensitivities to variable therapeutic drugs. Patients with no response to fluorouracil-based ACT had higher risk scores in both the GSE45404 **(W)** and GSE62080 **(X)** cohorts. ns: no significance; *p < 0.05; **p < 0.01; and ***p < 0.001.

Subsequent analysis investigated the expression of immune checkpoint genes in both high- and low-risk groups. The results demonstrated that the low-risk group displayed heightened expression patterns of immune checkpoint genes, including, but not limited to, *PD-1* (*PDCD1*), *PD-L1* (*CD274*), *LAG-3*, and *CTLA-4* **(Figure 9A, *p* < 0.05)**, demonstrating that patients with low-risk CRC could have a more favorable response to ICI treatment. The TIDE score can be used to predict the likelihood of immune evasion. The study findings indicated that there was no significant variation in the overall TIDE scores** (Figure 9B, *p* > 0.05)** or immune dysfunction scores **(Figure 9C, *p* > 0.05)** between the two groups. However, Higher immune exclusion scores in the high-risk group **(Figure 9D, *p* < 0.001)** suggested a greater chance of immune exclusion and a poor reaction to ICI blockade therapy. A comparison was made between the IPSs for the two risk groups to further explore the CRC patient response to different types of ICI blockade treatment. The IPSs of patients who did not receive ICI therapy were almost equal in the two risk groups **(Figure 9E)**, whereas low-risk patients who received *PD-1*/*PD-L1*/*PD-L2*, *CTLA-4*, or *CTLA-4* and *PD-1*/*PD-L1*/*PD-L2* blockade treatment had significantly greater IPSs **(Figures 9F-H, *p* < 0.01)**, indicating a better response to ICI therapy. The iMvigor210 cohort was used to validate the efficacy of the risk score in anticipating ICI responses. Patients with CR/PR were more likely to obtain a lower risk score, whereas patients with a high-risk score had a higher probability of having SD/PD **(Figure 9I, *p* < 0.05)**.

### Association of Risk Score with the IC_50_ of Therapeutic Medicines

We compared and analyzed the IC_50_ values of the treatment drugs in the two groups. The low-risk group exhibited lower IC_50_ values for 13 drug types** (Figures 9J-V, *p* < 0.001)**, including 5-fluorouracil. GSE45404 and GSE62080 contained information on CRC patient responses to fluorouracil-based ACT. The results demonstrated that NR patients had higher risk scores in the GSE45404 **(Figure 9W, *p* < 0.01)** and GSE62080 **(Figure 9X, *p* < 0.05)** cohorts, indicating that low-risk patients had a greater response to fluorouracil-based ACT.

### Identification and Validation of Key TRGs Using WGCNA and qRT-PCR

**Figure 10:**
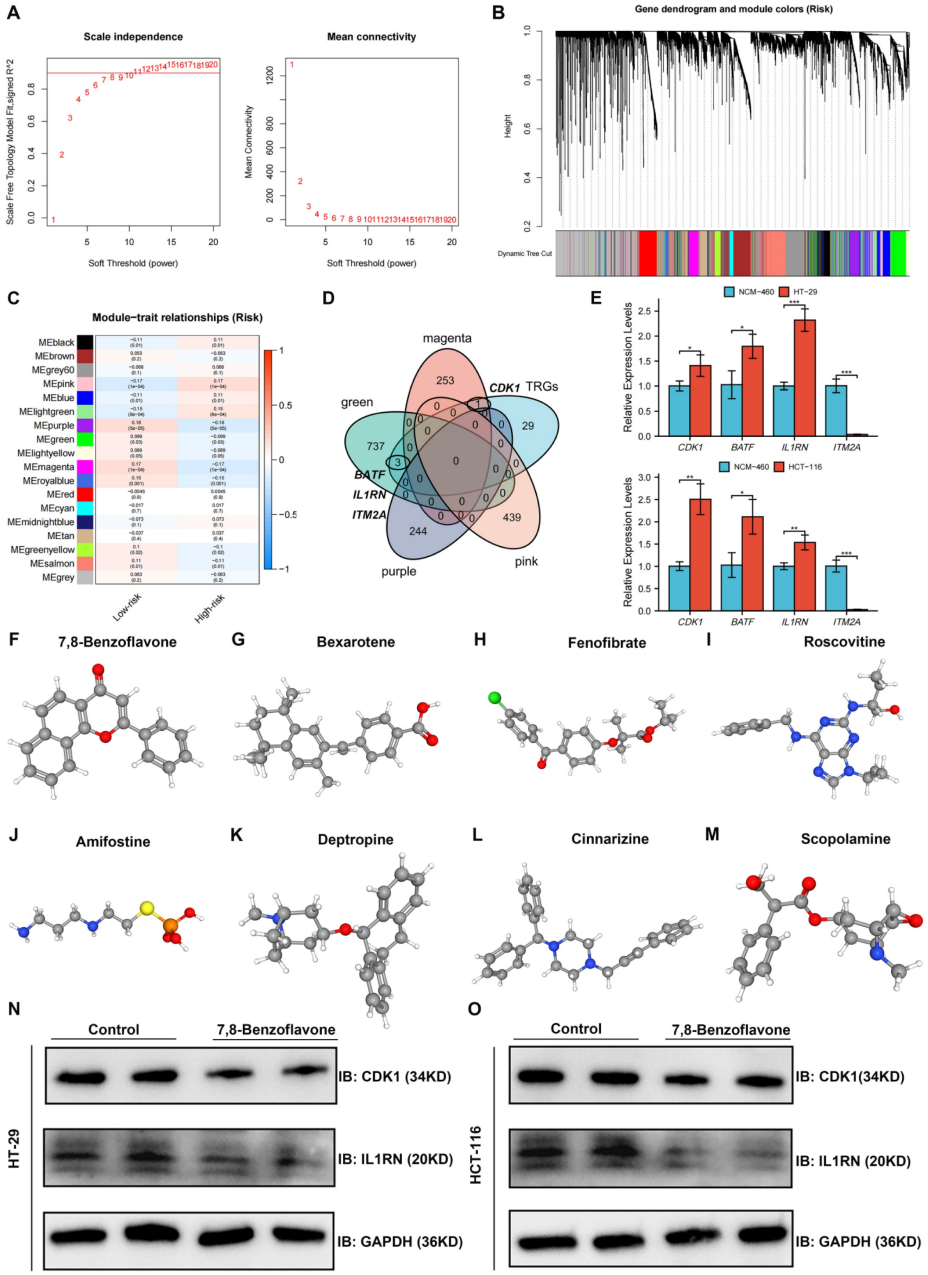
Identification and Validation of Key TRGs Using WGCNA and qRT-PCR, and screening potential therapeutic drugs based on key TRGs. **(A-B)** Seven was selected as the soft threshold, and 18 modules in total were obtained. **(C)** Nine modules were positively correlated with the risk score, whereas the other nine modules were negatively related to the risk score. **(D)** Intersection genes between the four most significant modules and the 33 TRGs were defined as key TRGs, and *CDK1*, *BATF*, *IL1RN*, and *ITM2A* were identified. **(E)** The *CDK1*, *BATF*, and *IL1RN* expression were higher in the HT-29 and HCT-116 cell lines than in the NCM-460 cell line, whereas *ITM2A* was significantly downregulated in the HT-29 and HCT-116 cell lines. The eight most significant drug molecules and their corresponding 3D structures are also presented, which are 7,8-benzoflavone **(F)**, bexarotene **(G)**, fenofibrate **(H)**, roscovitine **(I)**, amifostine **(J)**, deptropine **(K)**, cinnarizine **(L)**, and scopolamine **(M)**. 7,8-benzoflavone significantly decreased protein expression levels of *CDK1* and *IL1RN* in HT29 **(N)** and HCT116 **(O)** cell lines. *p < 0.05; **p < 0.01; and ***p < 0.001.

**Figure 11:**
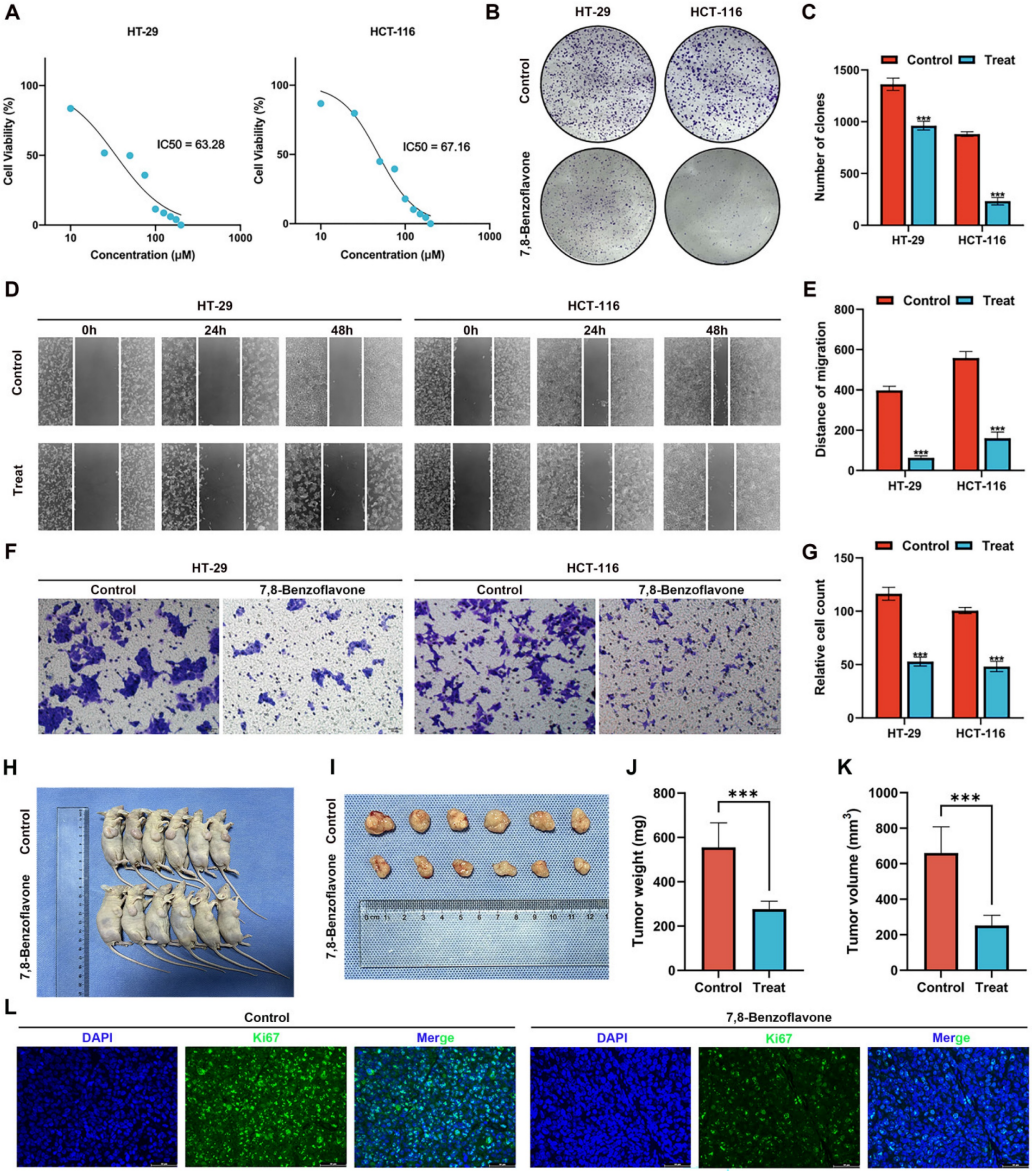
Using *in vitro* and *in vivo* experiments to validate the effects of 7,8-benzoflavone on CRC proliferation and migration. **(A)** The results of the MTT assay on HT-29 and HCT-116 CRC cells. **(B-C)** The experimental group showed decreased colony-forming abilities compared to the control group for both HT-29 and HCT-116 CRC cells. **(D-E)** Wound healing assay suggested that 7,8-benzoflavone decreased the migratory distance of CRC cells compared to that of cells treated with the control. **(F-G)** Transwell assay indicated that 7,8-benzoflavone significantly impaired the migration ability of CRC cells. **(H-I)** Images depicting tumor growth in the human CRC cells, HCT116 (2×10^6^ cells), injected into nude mice (n=6). **(J)** Tumor weights and **(K)** tumor volumes in different groups. **(L)** Immunofluorescence staining for *Ki67* in tumor tissues from nude mice with or without 7,8-benzoflavone treatment (Scale bar: 20 μm). ***p < 0.001.

Co-expression analysis using the WGCNA algorithm was performed to identify the key modules with the greatest relationship to the risk score. Seven modules were chosen as the soft threshold **(Figure 10A)**, and 18 modules were obtained in total** (Figure 10B)**. Nine modules were strongly related to the risk score, whereas the other nine modules were inversely related to the risk score **(Figure 10C)**. Intersection genes between the four most significant modules and 33 TRGs were defined as key TRGs, and *CDK1*, *BATF*, *IL1RN*, and *ITM2A* were identified** (Figure 10D)**. The four TRGs were mainly expressed in T cells, especially in proliferative T cells** (Figure S5)**. The expression of key TRGs in normal colon (NCM-460) and CRC (HT-29 and HCT-116) cell lines was validated using qRT-PCR, as shown in Figure 11E. *CDK1*, *BATF*, and *IL1RN* expression was higher in HT-29 and HCT-116 cells than in NCM-460 cells, whereas *ITM2A* was significantly downregulated in both cell lines** (Figure 10E)**.

### Screening Potential Therapeutic Drugs Based on Key TRGs

A list of drug molecules related to these four key TRGs is provided in **Table S4**. The eight most significant drug molecules and their corresponding 3D structures are presented: 7,8-benzoflavone **(Figure 10F)**, bexarotene** (Figure 10G)**, fenofibrate **(Figure 10H)**, roscovitine** (Figure 10I)**, amifostine** (Figure 10J)**, deptropine **(Figure 10K)**, cinnarizine **(Figure 10L)**, and scopolamine **(Figure 10M)**. Results of Western blot showed that 7,8-benzoflavone significantly decreased protein expression levels of *CDK1* and *IL1RN* in HT29 **(Figure 10N)** and HCT116 **(Figure 10O)** cell lines.

### Validating the Effects of 7,8-benzoflavone on CRC Proliferation and Migration via *in vitro* Experiments

The results of the MTT assay on HT-29 and HCT-116 CRC cells** (Figure 11A)** revealed that 7,8-benzoflavone significantly inhibited the viability of CRC cells. Based on the fitting formula, the IC_50_ values of 7,8-benzoflavone for HT-29 and HCT-116 were 63.28 μM and 67.16 μM at 48 h of therapy, respectively. The experimental group showed reduced colony-forming abilities compared with the control group for HT-29 and HCT-116 CRC cells **(Figures 11B-C)**. The findings of the wound healing assay suggested that 7,8-benzoflavone reduced the migratory distance of CRC cells compared to that of control-treated cells** (Figures 11D-E)**. Furthermore, the results of the transwell assay indicated that 7,8-benzoflavone significantly reduced CRC cell migration** (Figures 11F-G)**.

### Validating the Effects of 7,8-benzoflavone on CRC Proliferation via *in vivo* Experiments

BALB/c nude mice were subcutaneously injected with HCT116 cells to create xenograft tumors. Subsequently, mice were administered the vector or 7,8-Benzoflavone at 2-day intervals for a duration of 4 weeks once the tumors had attained an average volume of 100 mm^3^. The findings of our study demonstrated a significant reduction in tumor size** (Figure 11H-I)**, tumor weight **(Figure 11J)**, and tumor volume **(Figure 11K)**. Further analysis of tumor tissues revealed a noticeable decrease in the count of *Ki67* positive cells in 7,8-benzoflavone-treated mice compared to that in the control group** (Figure 11L)**. These findings indicate that 7,8-benzoflavone could potentially suppress the proliferation of CRC cells *in vivo* by downregulating *Ki67* expression.

## Discussion

In a previous study, 33 TRGs were identified using genome-scale screening 15. However, the ability of these TRGs to predict CRC patient prognosis and treatment sensitivity and the potential for screening new therapeutic biomarker candidates and drug molecules has not been examined. Among these TRGs, some have already been shown to be related to CRC; for example, *CXCL12* is inversely expressed and correlated with migration and invasion in CRC cell lines 31. *CLIC1* is significantly overexpressed in CRC tissues compared to normal tissues and could serve as a potential biomarker 32. Its overexpression of *RAN* is related to poor clinical outcomes in patients with CRC 33, and *CDK1* has been identified as a potential indicator of tumor recurrence in stage II colon cancer 34. The expression, genetic and transcriptional alterations, and predictive variables of the TRGs were examined. The findings indicated that the majority of these TRGs exhibited different expression levels in CRC and were correlated with patient survival.

Based on TRGs expression, patients with CRC were divided into two TRG clusters. TRG cluster B showed higher survival rates and immune cell infiltration values, and was positively related to CRC-related pathways, including the MAPK 35, JAK-STAT 36, and chemokine 37 signaling pathways. The response to anti-checkpoint blockade therapy can be influenced by tumor-infiltrating immune cells, and upregulation of some immunological checkpoint genes, such as *PD-1* and *CTLA-4*, can be due to tumor-infiltrating *CD4*+ T cells 38. The expression of *PD-1*, *PD-L1*, and *CTLA-4* in the TRG clusters was explored, and the results showed that these three immune checkpoints had higher expression in TRG cluster B than in cluster A. PRDEGs between the two TRG clusters were further determined, and the patients with CRC were split into three gene clusters based on PRDEGs expression. The prognoses of CRC patients in the three gene clusters were significantly different. Gene cluster A exhibited the lowest expression of *PD-1*, *PD-L1*, and *CTLA-4*, whereas immunological checkpoint expression was the highest in cluster C.

LASSO and stepwise Cox analyses were conducted to determine genes that could be utilized to construct the predictive signature, and 10 signature genes were identified and utilized to measure the risk score. Among these 10 signature genes, *KLF2* could inhibit cell growth in a human CRC cell line 39, *SCR2* was determined as a predictive biomarker and was associated with immunological cell infiltration in CRC 40, and *CXCL13* was associated with poor survival in advanced CRC patients and may regulate 5-fluorouracil resistance 41, 42. The risk score was used to classify the patients into two groups. The low-risk group exhibited a significant increase in the overall survival time relative to the high-risk group. These results also showed that the risk score has the potential to serve as a distinct predictive biomarker for CRC. The effectiveness of the risk score in predicting survival was assessed throughout multiple cohorts, and survival curves and ROC analysis indicated that the risk score had a consistent prediction ability in various cohorts. A meta-analysis showed no evidence of heterogeneity among the cohorts. The risk score was further compared with 10 published CRC signatures and revealed an improved efficiency in predicting patient survival. We also discovered that the risk score was strongly associated with angiogenesis and EMT in various tumor types, suggesting that an elevated risk score is typically associated with increased angiogenesis and increased aggressive tumor cells.

The TME is a critical factor in tumor development, progression, and drug resistance 43. The association of risk score with tumor-infiltrating immunological cells was measured. Ten forms of immunological cells were connected to the risk score, and the signature genes revealed strong correlations with different types of immune cells. The low-risk group exhibited a reduced stromal score, and immunogenomic analysis can provide an immune score that may serve as an indicator of the effectiveness of immunotherapy and chemotherapy 44, revealing that the risk score has the potential to be utilized to anticipate treatment response in patients with CRC. TMB levels in the low-risk group were significantly higher than those in the high-risk group, suggesting that immunotherapy may be more beneficial for patients with low CRC scores. MSI is attributed to various mismatch repair mechanisms that are significantly linked to the response to *PD-1* blockade treatment 45. The percentage weight of MSI status was determined for the two groups. CSCs constitute a distinct population of neoplastic cells that contribute to tumor metastasis, relapse, and resistance to therapeutic interventions. These cells have the same self-renewal and differentiation capacities as normal stem cells 46. The risk score correlated with the CSC index, suggesting that the risk score may be linked to the occurrence and development of CRC. To examine the feasibility of utilizing risk scores as a predictive tool for immunotherapy response, an investigation was carried out to compare the expression patterns of different immune checkpoints between the two groups. The outcomes indicated that the low-risk group displayed greater immunological checkpoint expression. Compared to the low-risk group, the high-risk group had a higher exclusion score, suggesting an increased possibility of immune escape. Furthermore, Patients categorized as low-risk exhibited a statistically significant increase in IPS compared to patients classified as high-risk who received ICI therapy. These results suggest that immunotherapy may be more efficient in low-risk patients. The iMvigor210 cohort was used to verify this finding, and the outcomes demonstrated that CR/PR patients had significantly decreased risk scores compared to patients with SD/PD, demonstrating that the risk score could effectively differentiate between cold and hot tumors and assist in precise CRC treatment mediation. The IC_50_ values of various therapeutic drugs were compared to determine their sensitivity to drugs in the two risk groups. The low-risk group had reduced IC_50_ values and exhibited greater sensitivity to the administered drugs, including 5-fluorouracil. We used the GSE45404 and GSE62080 cohorts to validate patient response to fluorouracil-based ACT. The results successfully validated our finding that patients who responded to fluorouracil-based ACT had lower risk scores.

The WGCNA algorithm was used to screen for key TRGs, among which *CDK1*, *BATF*, *IL1RN*, and *ITM2A* were identified as key TRGs. qRT-PCR was performed to determine the expression of the four key TRGs in the normal human colon and CRC cell lines. *CDK1*, *BATF*, and *IL1RN* were upregulated in CRC cell lines compared to that in normal colon cell lines, whereas *ITM2A* showed significantly lower expression in CRC cells. Based on the four TRGs, a list of potential therapeutic drug molecules was screened and the 3D structures of the eight most significant drugs were determined. 7,8-benzoflavone, also called alpha-naphthoflavone, was identified as the most significant drug molecule. Human T cells are extremely sensitive to inhibition of mitogenesis by polycyclic aromatic hydrocarbons, and 7,8-benzoflavone can counteract this effect 47. In addition, 7,8-benzoflavone can suppress proliferation and induce apoptosis in human cervical cancer cells (HeLa cells) 48. It can also hinder colon cancer clonogenicity 49. To further explore the effects of 7,8-benzoflavone on CRC, MTT, wound healing, Transwell, colony formation assays, and in-vivo experiments were performed. The results indicated that 7,8-benzoflavone could inhibit the proliferation and migration of CRC cells, indicating its potential as a drug molecule for CRC therapy.

Our study was subject to certain constraints. First, our analysis was conducted using publicly available datasets and retrospectively gathered samples, which could have resulted in an inherent selection bias. Second, the mechanisms by 7,8-Benzoflavone affects CRC proliferation and migration require further exploration. Finally, some meaningful clinical characteristics, including surgery and tumor markers, were excluded from this investigation. Therefore, additional clinical studies are required to validate our findings.

## Conclusion

Overall, T-cell proliferation-based molecular subtypes and predictive models can be utilized to predict patient outcomes, immunological landscape, and treatment sensitivity to CRC. Novel biomarker candidates and potential therapeutic drugs were identified and verified by *in vitro* experiments. However, additional *in vivo* experiments should be performed and clinical cases should be collected to further confirm our results.

## Supplementary Material

Supplementary figures and tables.

## Abbreviations

TRGs: T cell proliferation-related gene
CRC: Colorectal cancer
DEGs: Differentially expressed genes
PD-1: Programmed cell death protein 1
FDA: Food and Drug Ministration
ICIs: Immune checkpoint inhibitors
dMMR: Deficient mismatch repair
MSI: Microsatellite instability
TMB: Tumor burden mutations
NAL: Neoantigen load
TME: Tumor microenvironment
NK: Natural killer
TCGA: The Cancer Genome Atlas
GEO: Gene Expression Omnibus
HNSCC: Head and neck squamous cell carcinoma
IFN: Interferon
PD-L1: Programmed cell death 1 ligand 1
OS: Overall survival
ACT: Adjuvant chemotherapy
FPKM: Fragments per kilobase million
TPM: Transcription per million
GO: Gene Ontology
KEGG: Kyoto Encyclopedia of Genes and Genomes
PCA: Principal component analysis
GSVA: Gene set variation analysis
ssGSEA: Single-sample gene set enrichment analysis
CTLA-4: Cytotoxic T-lymphocyte-associated protein 4
PRDEGs: Prognosis-related DEGs
LASSO: Least absolute shrinkage and selection operator
ROC: Receiver operating characteristic
EMT: Epithelial to mesenchymal transition
TMB: Tumor Mutation Burden
CSC: Cancer Stem Cell
TIDE: Tumor Immune Dysfunction and Exclusion
IPS: Immune Cell proportion Score
CR: Complete response
PR: Partial response
SD: Stable disease
PD: Pogressive disease
NR: Non-response
R: Response
AM: adjacency matrix
cDNA: Complementary DNA
DSigDB: Drug Signatures Database
3D: Three-dimensional
DMEM: Dulbecco's modified Eagle's medium
DMSO: Dimethyl sulfoxide
PBS: Peripheral blood smear
SPF: Specific pathogen-free
CNV: Copy number variation
BP: Biological processes
CC: Cellular components
MF: Molecular functions
AUC: Area under curve
