# Supplementary material for: Identification of novel T cell proliferation patterns, potential biomarkers and therapeutic drugs in colorectal cancer
Source: J Cancer. 2024 Jan 1;15(5):1234–54. doi: 10.7150/jca.91835 (PMC10861827; doi:10.7150/jca.91835)
Supplement: Supplementary file 1 — Supplementary figures and tables. [file jcav15p1234s1.zip › Supplemental files/legends.pdf]

**Supplemental files**

**Table S1:** Clinical information of patients in all datasets.

**Table S2:** List of 33 TRGs.

**Table S3:** Coefficient values of 10 signature genes in the multivariate Cox regression analysis.

**Table S4:** A list of the drug molecules related to four key TRGs.

**Figure S1:** The somatic mutation incidence of TRGs in patients with CRC. COAD: colon adenocarcinoma; READ: rectum adenocarcinoma.

**Figure S2:** Survival curves of prognosis-related TRGs.

**Figure S3:** The expression of the TRGs were used to perform a consensus clustering analysis to classify patients into two TRG clusters.

**Figure S4:** Patients with CRC were divided into three gene clusters using consensus clustering.

**Figure S5:** Expression of key TRGs in GSE108989 and GSE146771 single cell datasets using TISCH database.
