# Supplementary figures and images for: Identification of novel T cell proliferation patterns, potential biomarkers and therapeutic drugs in colorectal cancer
Source: J Cancer. 2024 Jan 1;15(5):1234–54. doi: 10.7150/jca.91835 (PMC10861827; doi:10.7150/jca.91835)

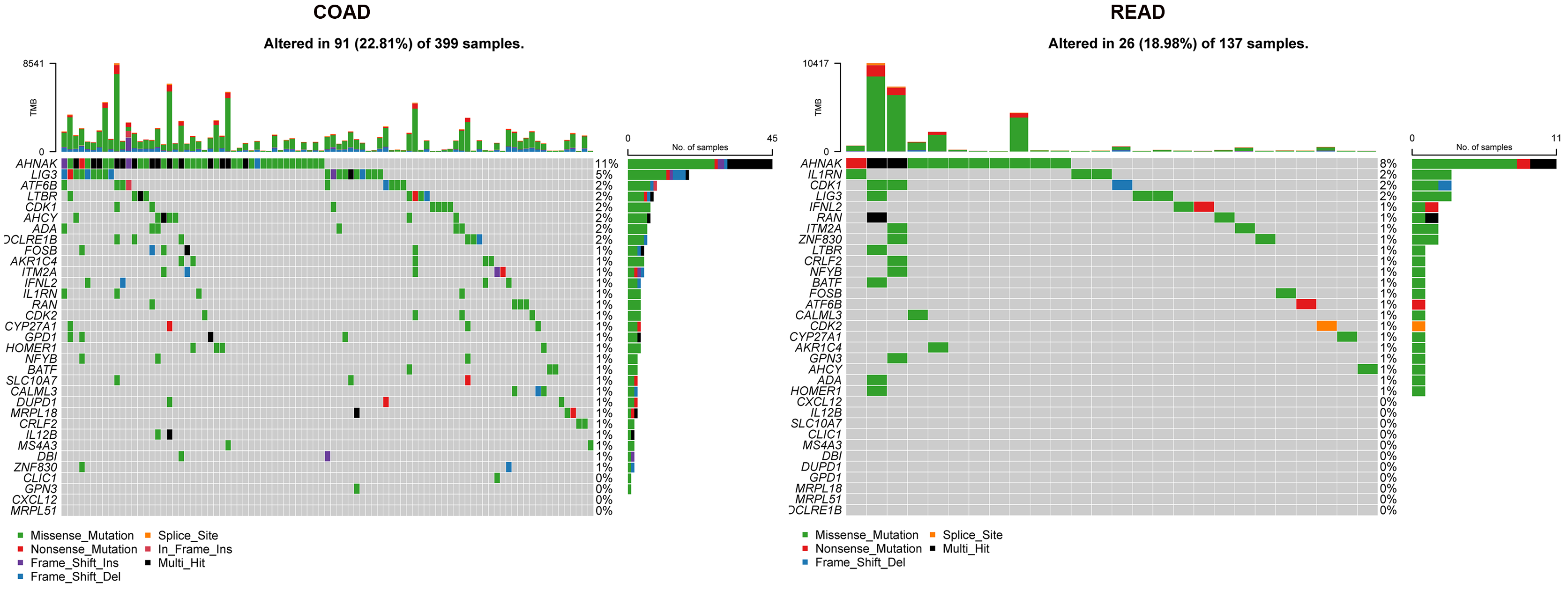

Supplement: Supplementary file 1 — Supplementary figures and tables. [file jcav15p1234s1.zip › Supplemental files/Figure S1.tif]

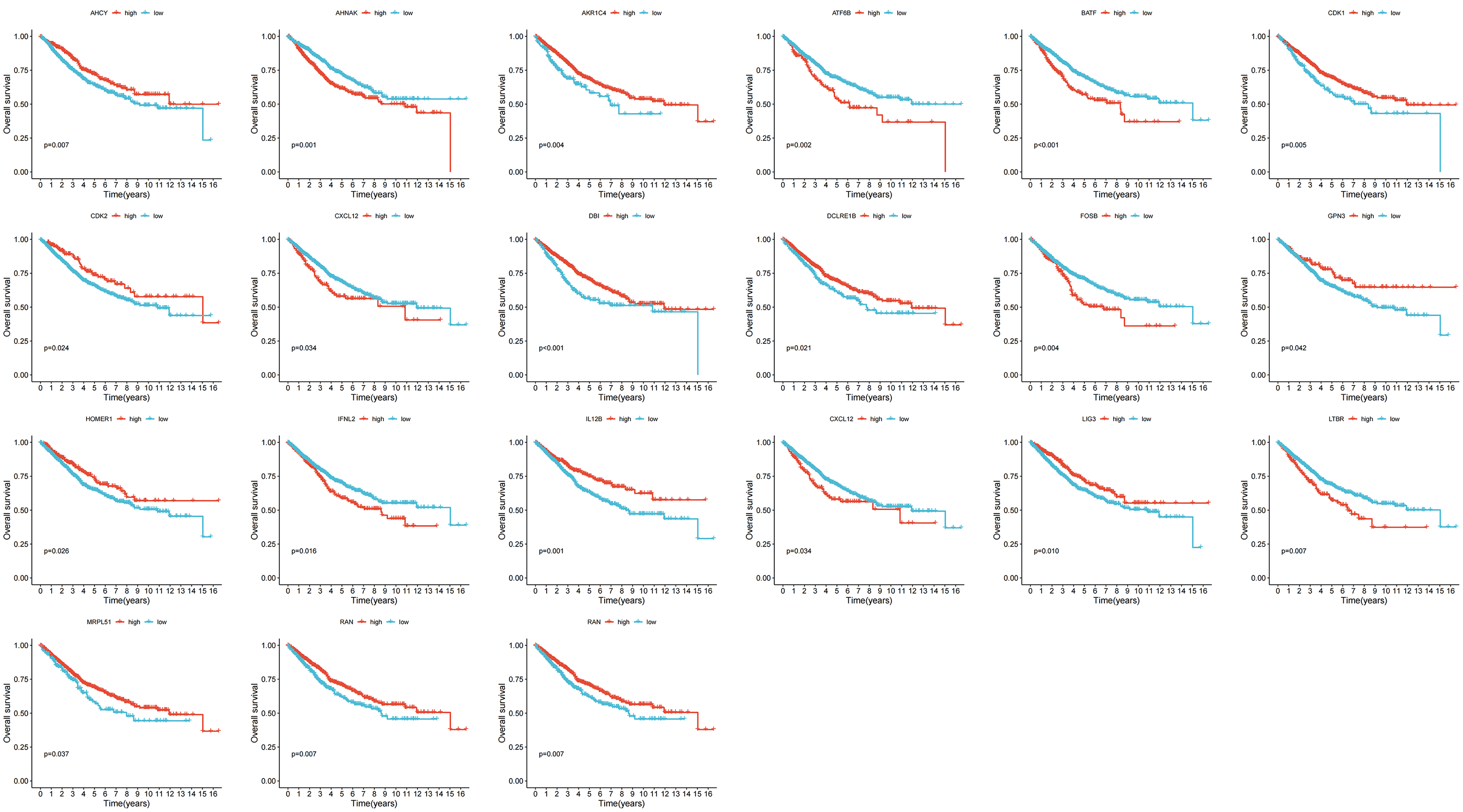

Supplement: Supplementary file 1 — Supplementary figures and tables. [file jcav15p1234s1.zip › Supplemental files/Figure S2.tif]

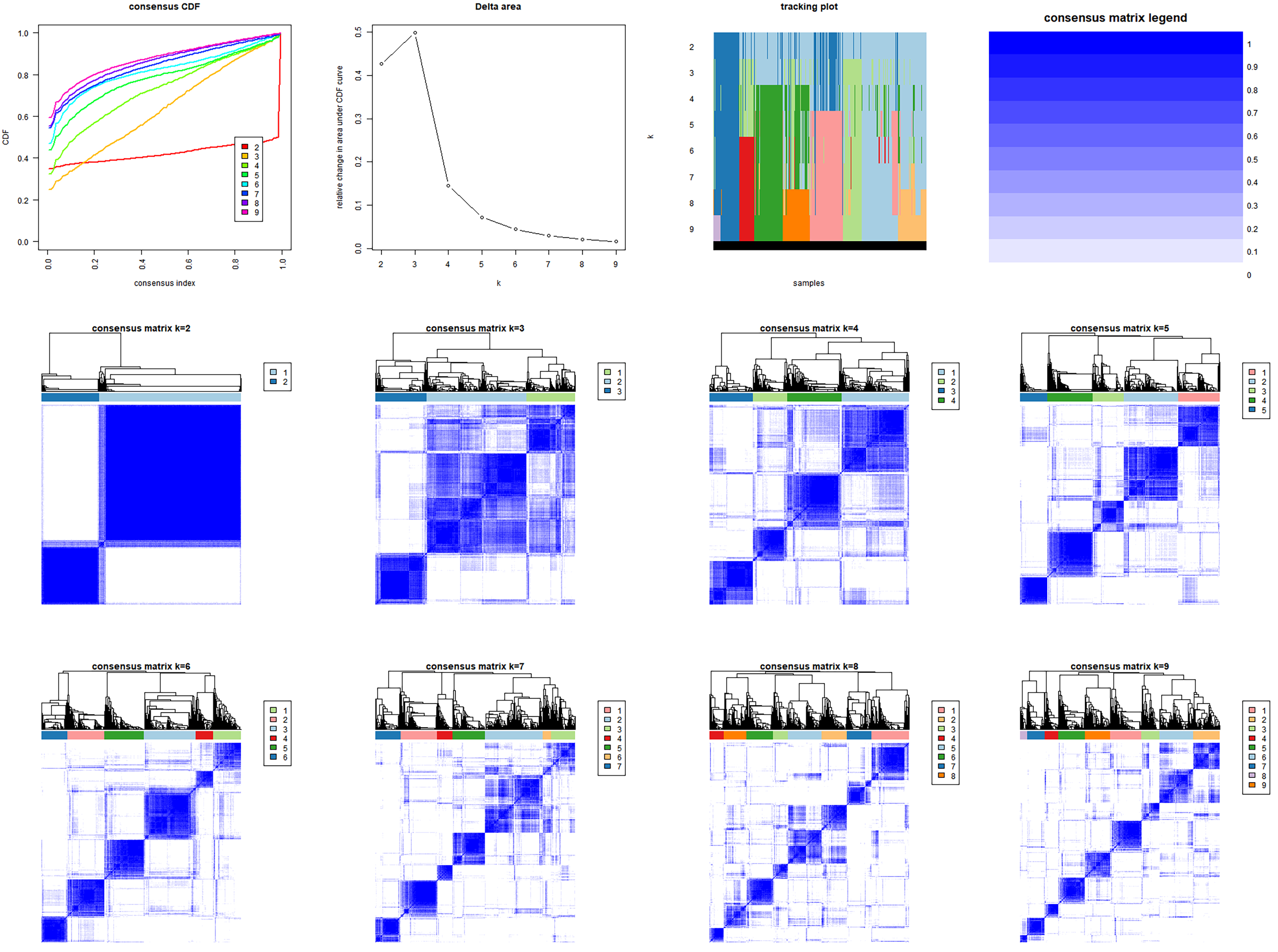

Supplement: Supplementary file 1 — Supplementary figures and tables. [file jcav15p1234s1.zip › Supplemental files/Figure S3.tif]

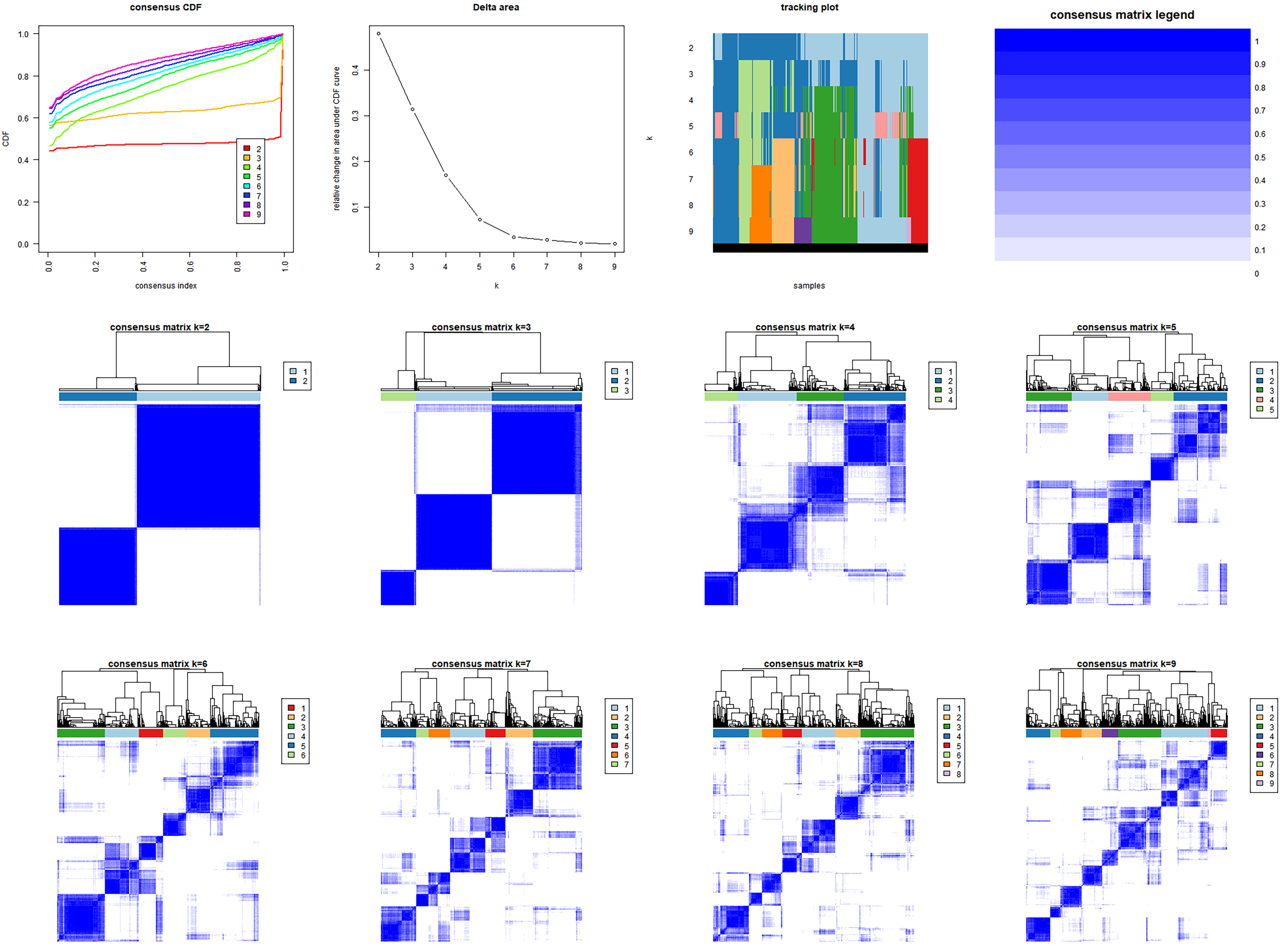

Supplement: Supplementary file 1 — Supplementary figures and tables. [file jcav15p1234s1.zip › Supplemental files/Figure S4.tif]

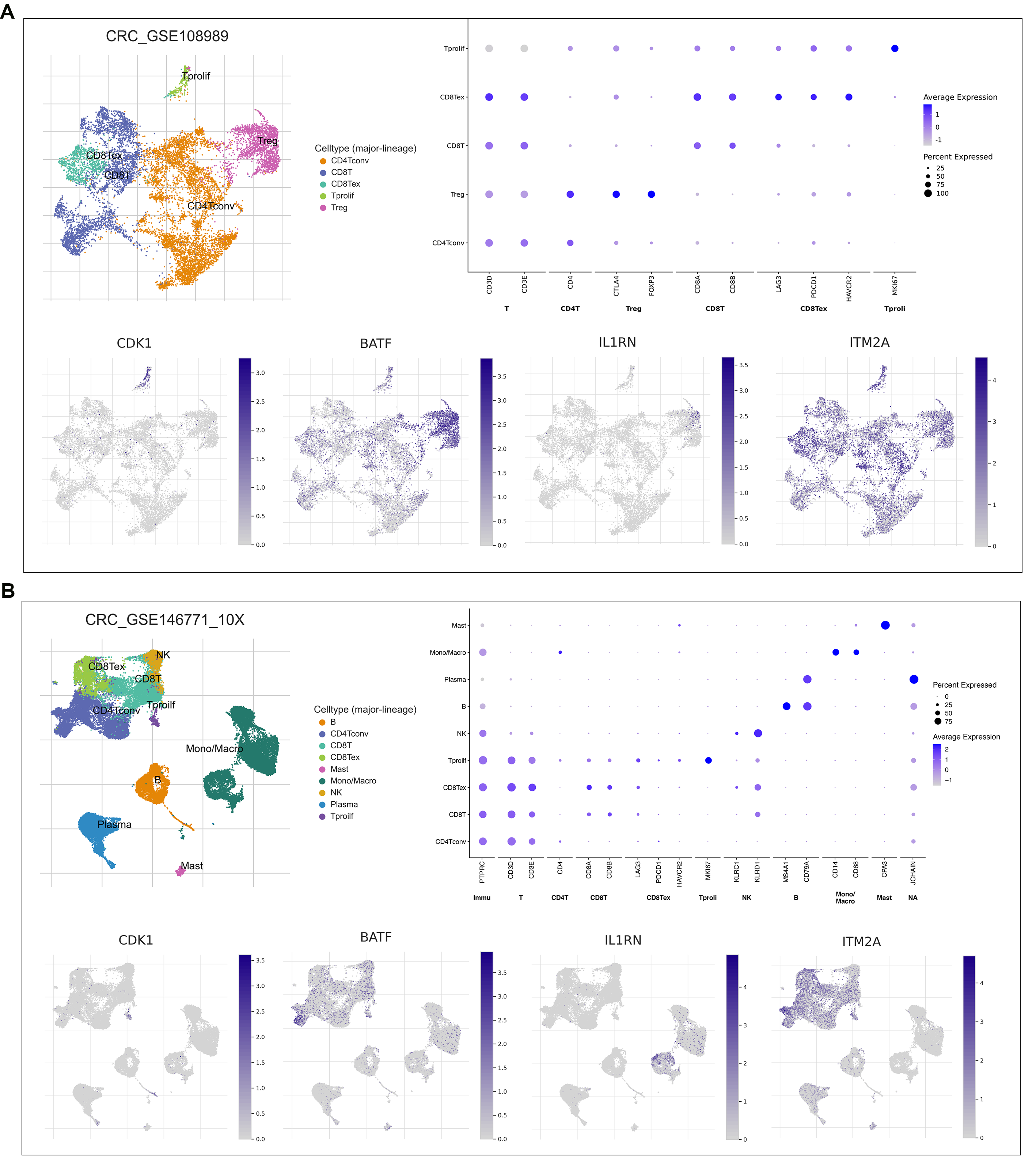

Supplement: Supplementary file 1 — Supplementary figures and tables. [file jcav15p1234s1.zip › Supplemental files/Figure S5 .tif]
